# The specious art of single-cell genomics

**DOI:** 10.1371/journal.pcbi.1011288

**Published:** 2023-08-17

**Authors:** Tara Chari, Lior Pachter

**Affiliations:** 1 Division of Biology and Biological Engineering, California Institute of Technology, Pasadena, California, United States of America; 2 Department of Computing and Mathematical Sciences, California Institute of Technology, Pasadena, California, United States of America; University of Virginia, UNITED STATES

## Abstract

Dimensionality reduction is standard practice for filtering noise and identifying relevant features in large-scale data analyses. In biology, single-cell genomics studies typically begin with reduction to 2 or 3 dimensions to produce “all-in-one” visuals of the data that are amenable to the human eye, and these are subsequently used for qualitative and quantitative exploratory analysis. However, there is little theoretical support for this practice, and we show that extreme dimension reduction, from hundreds or thousands of dimensions to 2, inevitably induces significant distortion of high-dimensional datasets. We therefore examine the practical implications of low-dimensional embedding of single-cell data and find that extensive distortions and inconsistent practices make such embeddings counter-productive for exploratory, biological analyses. In lieu of this, we discuss alternative approaches for conducting targeted embedding and feature exploration to enable hypothesis-driven biological discovery.

## Introduction

**Fig 1:**
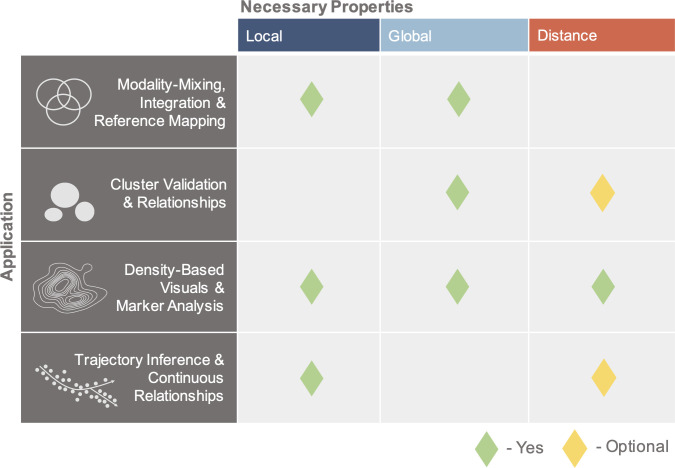
Necessary properties for embedding applications. Application rows denote biological tasks, and columns denote which properties are necessary, i.e., key geometric properties whose preservation or representation is assumed in the task.

The high-dimensionality of “big data” genomics datasets has led to the ubiquitous application of dimensionality reduction to filter noise, enable tractable computation, and to facilitate exploratory data analysis (EDA). Ostensibly, the goal of this reduction is to preserve and extract local and/or global structures from the data for biological inference [1–3]. Trial and error application of common techniques has resulted in a currently popular workflow combining initial dimensionality reduction to a few dozen dimensions, often using principal component analysis (PCA), with further nonlinear reduction to 2 dimensions using t-SNE [4] or UMAP [1,2,5,6]. For single-cell genomics in particular, these embeddings are used extensively in qualitative and quantitative EDA tasks that fall into 4 main categories of applications (Fig 1, “Application”):

- Modality-mixing, integration, and reference mapping:

Embeddings are used to visually assess the extent of integration, mixing, or similarities between cells from different batches [7–9] and to compare methods of integration/batch-correction [10]. For query dataset(s) mapped onto reference datasets/embeddings, visuals likewise provide an assessment of merged data similarities or differences [11,12].

- Cluster validation and relationships:

Visual applications range from assessing the existence of and relationships between predefined clusters, to inferring properties of the clusters (e.g., spread or heterogeneity) [1,2,13], and to generating the clusters themselves from the 2D space (e.g., to define cell types or to detect doublets) [3,14,15].

- Density-based visuals and marker analysis:

Embeddings are used to justify or measure changes in cell populations between different conditions, by comparing contour locations and sizes in the density diagrams, as well as changes in intensity or spread of gene expression [16–20].

- Trajectory inference and continuous relationships:

Embedding applications range from implying or inferring local, continuous relationships between cells and assigning pseudotime coordinates [21–24], to using the 2D coordinates for explicit calculations of magnitude and direction of developmental progression [23,25,26].

Inherent in these applications are assumptions of preservation of local and global cell properties, as well as distances, delineated in Fig 1. For each application, we demarcate which of these are the “necessary” or key geometric properties that each task inherently assumes to be represented (and preserved). Based on previous works [6,13,27,28] and the objective functions of UMAP and t-SNE [4,5], “local” is defined as nearest neighbor relationships, “global” as neighbor relationships and properties of groups of cells (e.g., cell types), and “distance” as Euclidean distance (*L*_2_ norm) or Manhattan distance (*L*_1_ norm) between points. Note that preservation of distance implies preservation of local and global properties. We utilize the *L*_2_ norm as it is the default metric of UMAP/t-SNE. We also present results with the *L*_1_ norm (see S1 Text), as *L*_1_ is more suitable for measuring distance in high dimensions, particularly in comparison to other *L*_*k*_ norms [29,30], and is commonly applied to transcriptomic data [31–33], with comparable performance to the probabilistic Jensen–Shannon divergence in single-cell distance calculations [34].

Yet, despite the goals of these methods [2,3,6] to preserve local and/or global structure, there is little theory or empirical analysis to support these claims. For example, while the popular t-SNE and UMAP methods claim faithful representation of local and/or global structure in low dimensions [1,2,5], there is evidence they fail in this regard [1,35], and theorems providing guarantees on the embeddings rely on numerous assumptions unlikely to hold in practice and ignore the preprocessing by PCA prior to nonlinear reduction [36].

Here, we assess dimensionality reduction for single-cell gene expression, first investigating the preservation of the necessary properties comprising the columns of Fig 1, then assessing the impact of these embeddings across the applications comprising the rows of Fig 1.

## Preservation of local and global structure in 2D embeddings

We begin with the columns of Fig 1, and assess the preservation of these properties by 2D embedding, as compared to the ambient space or higher-dimensional PCA space to which the ambient space is initially reduced prior to reduction to 2D (see Methods in S1 Text).

“Ambient” space refers to the gene count matrix after highly variable gene selection and log-normalization of the counts (see Methods in S1 Text). We denote “PCA-preprocessing” as the higher dimensional reduction of the ambient space by PCA, followed by a (nonlinear) reduction to 2D (e.g., “PCA-50D→UMAP”) which mimics standard practice. Additionally, cell annotations or labels (such as cell type or condition) used in the following analyses were taken from the original studies.

### Local preservation

Given the focus on preserving local nearest neighbors in the objectives of the UMAP and t-SNE methods, we first measured the recapitulation of nearest neighbors in 2D embeddings, as compared to the neighbors defined in ambient space. We used Euclidean (*L*_2_) distance, the default for these nonlinear reduction methods, to define each cell’s 30 nearest neighbors and measured Jaccard distance (dissimilarity) between the neighbors in embedding and ambient space (where 1.0 denotes no overlap). Several in vivo datasets were reduced to 2D, with PCA-preprocessing, including 10× Genomics and SMART-Seq assayed mouse ventromedial hypothalamus (VMH) neuron datasets [37], an ex utero cultured mouse embryo dataset (at the E8.5 stage) and an ex and in utero mouse embryo dataset (at the E10.5 stage) from [8], and a mouse primary motor cortex (MOp) dataset [38]. We additionally reduced cell culture-derived datasets, with and without external perturbations, including mouse embryonic stem cells (mESCs) treated in DMSO from [39] and multiplexed mouse neural stem cells (NSCs) in 96 drug combination conditions (labeled “96-plex”) [40] (see Table A in S1 Text).

**Fig 2:**
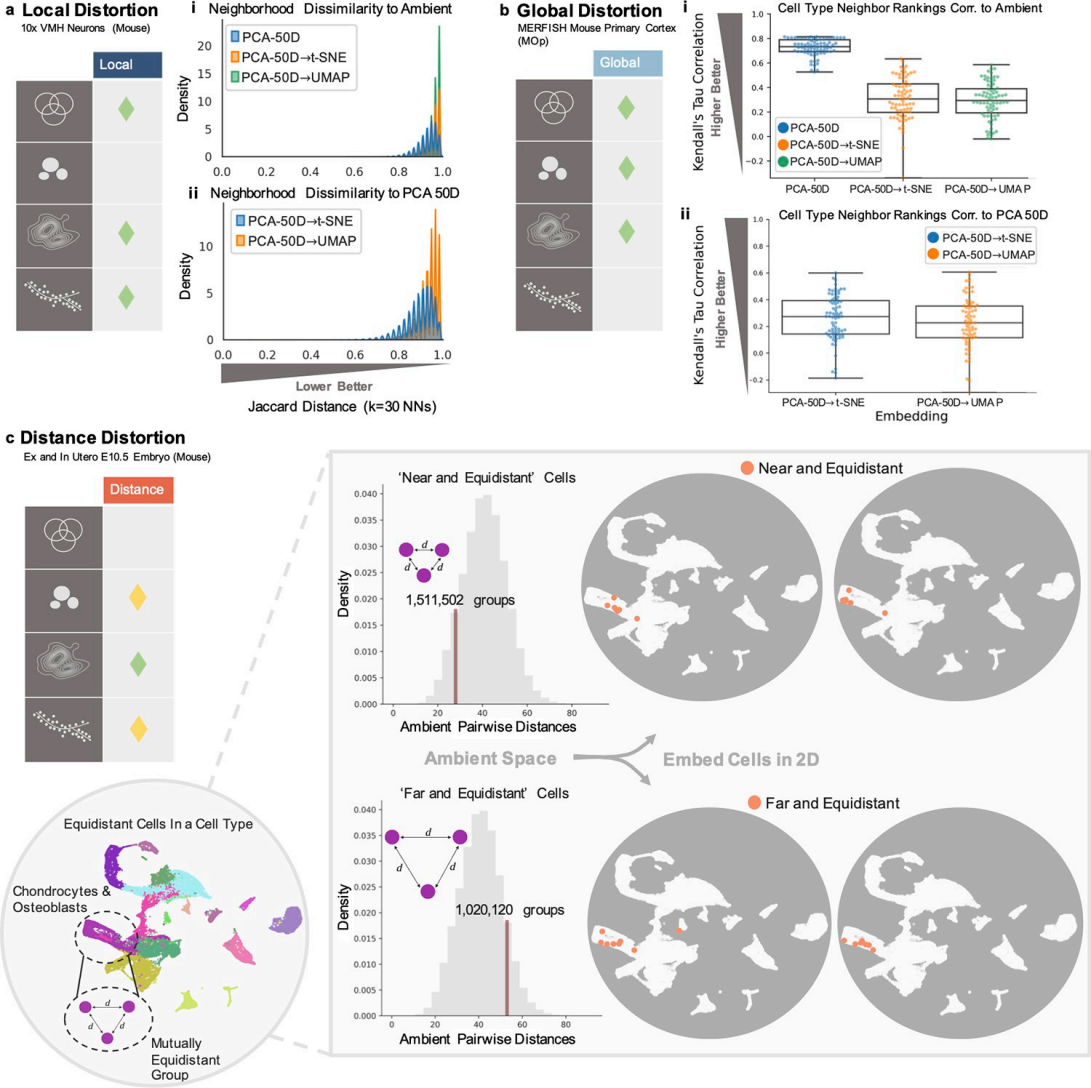
Distortion of necessary properties in embeddings. (**a**) (i) Distribution of Jaccard distance of cell neighbors in PCA-preprocessed 2D embeddings and the relevant PCA space, as compared to ambient space. (ii) Distribution of Jaccard distance of cell neighbors in PCA-preprocessed 2D embeddings, as compared to the higher dimensional PCA space. (**b**) (i) Boxplot of correlations of cell type neighbor rankings to ambient space for the PCA-preprocessed 2D embeddings and the relevant PCA space. (ii) Boxplot of correlations of cell type neighbor rankings to the relevant higher dimensional PCA space for the PCA-preprocessed 2D embeddings. Embeddings generated *n* = 3 times. (**c**) Selection of equidistant groups with “near” or “far” distances in ambient space. UMAP embedding of the data in gray circles, with orange circles denoting all cells within the previously determined equidistant groups.

The 2D t-SNE/UMAP embeddings (e.g., “PCA-50D→UMAP” in Fig 2A) displayed large Jaccard distances with respect to the neighbors in ambient dimension, with an average consistently above 0.7 (70%). Generally, dissimilarity increased with the size of the dataset (Fig 2A, Figs A and Ba in S1 Text). When the number of neighbors (k), considered in the dissimiliarity calculation, was varied between 5 to 100, smaller dataset embeddings displayed slightly improved dissimilarity scores with larger k (Figs Bb and Bc in S1 Text). Interestingly, the embeddings of the more homogeneous mESCs dataset displayed relatively higher dissimilarity despite the small number of cells (Figs Bb and Bc in S1 Text). Poor neighborhood overlap was additionally retained, and often worsened, without PCA-preprocessing (i.e., direct reduction to 2D from ambient space). In some cases, the dissimilarity of neighbors was worse for 2D PCA (“PCA-2D”) as compared to t-SNE or UMAP reduction without PCA-preprocessing, consistent with other findings on the poor preservation of local neighborhoods by both PCA and the nonlinear reduction methods [1,35] (Figs A and Bc in S1 Text). Similarly poor neighbor retention from the ambient space was observed in the higher dimensional PCA spaces as well (“PCA-50D” Fig 2Ai, Figs A and B in S1 Text) [35], particularly for larger datasets. Even between the PCA-preprocessed 2D embeddings and their corresponding PCA space, Jaccard distances were consistently above 0.8 on average, regardless of the dimension of the initial PCA reduction (Fig 2Aii, right panels Fig A in S1 Text).

### Global preservation

Turning to global relationships, we measured the preservation of the rankings of neighbors of cell “types” rather than individual cells. Cell “types” denote either author-provided cell type (Fig 2Bii) or cell condition annotations. Rankings were constructed from average pairwise distances between the cells of the different types, across replicate 2D embeddings (see Methods in S1 Text). For the same datasets as above, and a multiplexed dataset of human monocytes treated with 40 drugs [41], correlation of cell type neighbor rankings to that of the ambient space were low (≤ 0.4) in PCA-preprocessed 2D embeddings, and at least 33% lower than those of the higher dimensional PCA spaces, with warped or even reversed correlations in comparison to the ambient (Fig 2Bi) or relevant PCA space (Fig 2Bii, Fig Ca in S1 Text). These distortions were not specific to the distance measure used; we observed similar results when using the *L*_1_ norm to determine cell type neighbors (Fig Cb in S1 Text). This is consistent with observations made in other studies [6,28]. In general, correlation decreased over each step in the reduction process though there was not a clear trend related to other dataset properties (Figs Da and Ea in S1 Text). For analyses of recapitulation of cluster properties such as inferred heterogeneity or spread, see “Clustering validation and relationships” and “Embedding properties are arbitrary” below.

### Distance preservation

To examine distance preservation, we extracted groups of cells with quantitatively distinct relationships in the ambient space of the Seurat-integrated [7] ex and in utero mouse embryo dataset (at the E10.5 stage) [8], specifically equidistant groups of cells, where the distances between cells were all either equally small (“near”) or large (“far”) (Fig 2C) (see Methods in S1 Text). This revealed upwards of 2.5 million such groups, with 3 to 8 cells in each (Figs Fa and Fe in S1 Text). However, once embedded into 2 dimensions, these quantitatively distinct groups of cells (orange dots on UMAPs, Fig 2C) displayed the same dispersion patterns, violating distance preservation, and rendering these distinct, transcriptomic relationships indistinguishable.

This is not surprising, given previous theoretical work on the limits of distance preservation in low dimensions, particularly for equidistant points [42–44]. The Johnson–Lindenstrauss lemma on the optimality of linear embedding [45–47] shows that preservation of pairwise distances with a margin of error of at most 20% for a modestly sized dataset of 10,000 cells would require at least 1,842 dimensions [48]. Distortion is inevitable: given *n* points embedded in 2 dimensions, the distortion of the ratio of their maximum distance, *D*, to minimum distance, *d* (“max/min ratio”), grows as $O(\sqrt{n})$ [49] (see Note in S1 Text).

In practice, measuring these “max/min ratios” in 2D embeddings, for the ex and in utero data (E10.5) as well as the 10× VMH neurons, revealed 4- to 200-fold increases in these ratios whether compared to the relevant PCA space or ambient space (with or without PCA-preprocessing). This was the case in groups of equidistant cells as well as groups of nearest neighbors (Figs F and G in S1 Text) and can result in trends such as displayed in Fig 2C, with cells shot out across the embedding. For both datasets, we empirically verified the growth of this distortion with the number of cells considered in each equidistant group, i.e., as more cells are considered in 2D, the distortion grows (Fig H in S1 Text). Higher dimensional PCA spaces largely maintained similar max/min ratios to the ambient space (Figs G and H in S1 Text). However, we note that in low dimensions PCA embedding of equidistant points is tantamount to applying a random projection, similarly resulting in projected points displaying numerous mirages of structure or outliers (Fig I in S1 Text).

## Distortion of trends in applications

Given the distortions of the necessary properties in Fig 1, we then investigated their impact on each row or application, i.e., how in practice such embeddings affect the inferences and implications made in each application.

### Modality-mixing, integration, and reference mapping

**Fig 3:**
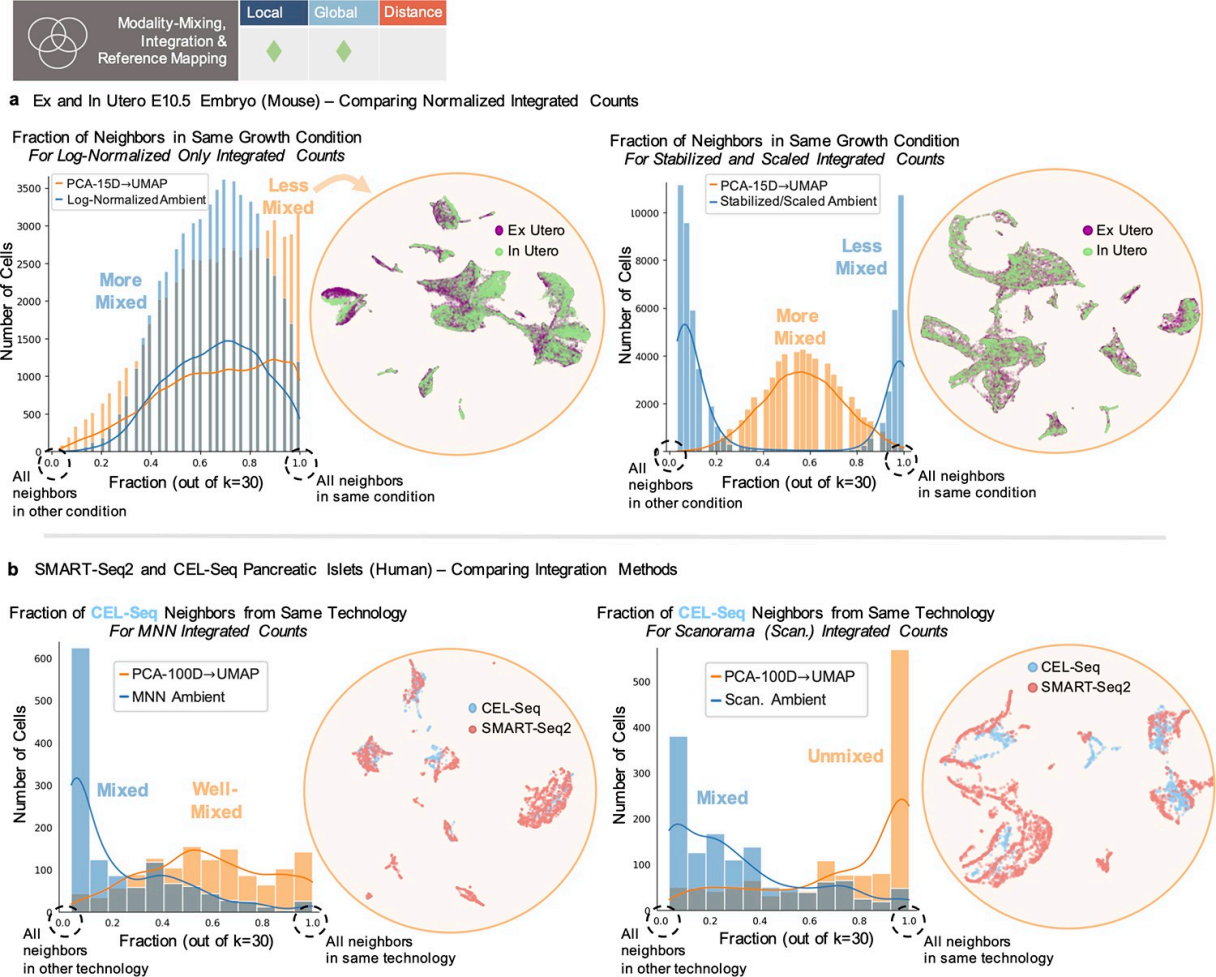
Distortion of mixing patterns. (**a**) Left plot shows “Log-normalized” ambient (blue) and 2D embedding (orange) distributions of mixing (fraction of cell neighbors in the same condition), where 1.0 is no mixing. Corresponding UMAP shown next to it. Right plot shows “Variance-Stabilized and Scaled” ambient (blue) and 2D embedding (orange) distributions of mixing (fraction of cell neighbors in the same condition). Corresponding UMAP shown next to it. (**b**) Left plot shows “MNN Integrated” ambient (blue) and 2D embedding (orange) distributions of mixing (fraction of cell neighbors in the same condition) for CEL-Seq cells. Corresponding UMAP shown next to it. Right plot shows “Scanorama Integrated” ambient (blue) and 2D embedding (orange) distributions of mixing (fraction of cell neighbors in the same condition) for CEL-Seq cells. Corresponding UMAP shown next to it.

Malleability of “structure” under low-dimensional embedding is particularly apparent in the mixing properties of integrated, mapped, or batch-corrected datasets, where an integration procedure is accompanied by an embedding of the melded datasets (Fig 3, Fig J in S1 Text) [7,8]. This relies on preserving both local relationships (which cells are mixed) and global patterns (overall trends of mixing or non-mixing between datasets) (Fig 1). For the integrated ex and in utero dataset (E10.5), we calculated the fraction of each cell’s nearest neighbors with the same label as the cell, to compare whether embeddings accurately reflect the extent of mixing of ex and in utero cells by integration (Fig 3A) (see Methods in S1 Text).

The “Log-Normalized” integrated, ambient data displayed a largely unimodal, well-mixed distribution of cells between conditions, while the distribution generated from embedding into 2 dimensions was shifted towards unmixed (left side, Fig 3A). The “Variance-Stabilized and Scaled” integrated, ambient data (a separate scaling procedure performed after integration) displayed the opposite trend. The ambient data presented a bimodal distribution with completely unmixed cell populations, while the final embedding displayed a unimodal distribution of well-mixed cells from both conditions (right side, Fig 3A). These additions or losses of mixing properties by 2D embedding were replicated using the *L*_1_ metric for neighbor determination (Fig J in S1 Text).

Such mixing patterns are not only used to argue that different datasets are similar, but also to argue for the superiority of one integration method over another. To assess whether such inferences are legitimate, we merged the SMART-Seq2 and CEL-Seq pancreatic islet datasets utilized in [10] with one of 2 methods, MNN [50] or Scanorama [10]. Looking at the fraction of mixing of CEL-Seq cells in the merged ambient space reveals similar mixing by both methods (CEL-Seq cells “mapped” to SMART-Seq2 cells) (ambient distributions, Fig 3B). However the UMAP embeddings provide opposite pictures, with MNN appearing to result in a well-mixed distribution of CEL-Seq cells (left side, Fig 3B) and Scanorama an unmixed distribution of cells (right side, Fig 3B). In cases where batch correction largely fails (Fig Kb in S1 Text), the “integrated” ambient spaces (by either method) are similar to the pre-integrated ambient space. However, reduction to 2D can enhance mixing for the “integrated” spaces, but decrease mixing in the pre-integrated space. We found similar distortions when the *L*_1_ norm was used and with t-SNE as used in [10] (Figs Jb, Jc, and Ka in S1 Text). Notably, the initial PCA reduction can drive the reversal or distortion of mixing trends, though removal of PCA-preprocessing does not alleviate this issue (Figs Jc and Ka in S1 Text). Thus, for a user, it is unclear what patterns of mixing are a result of the efficacy of the integration method, or arbitrary variation introduced by the dimensionality reduction procedure.

A consequence of these findings is that reference mapping procedures, which aim to demonstrate shared structures between batches or datasets, can also result in appearance of false structures (Fig L in S1 Text). As an example, UMAP has been proposed as a method for transforming or mapping new data given coordinates fit on another dataset [11]. Yet, transforming high dimensional, uniformly distributed points with UMAP coordinates from a single-cell dataset imposes a false structure akin to the structure of the single-cell data (Fig L in S1 Text) (see Methods in S1 Text).

### Cluster validation and relationships

**Fig 4:**
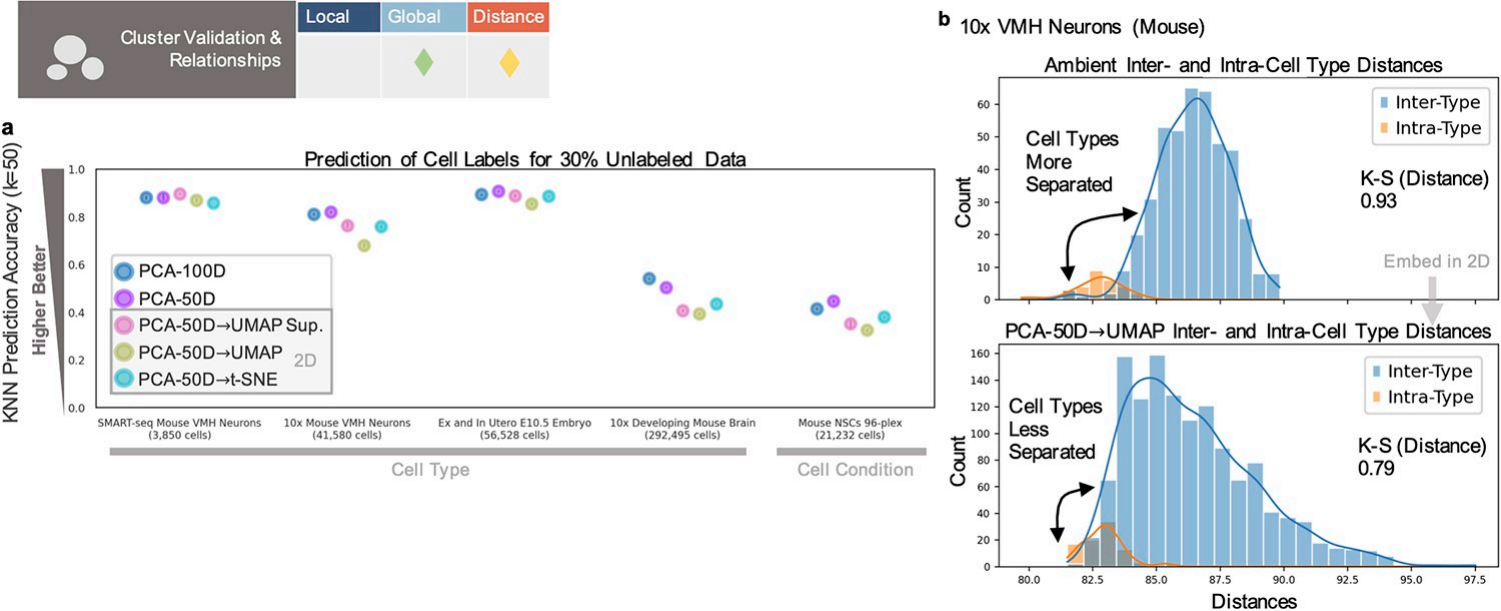
Distortion in cluster validation and relationships. (**a**) Prediction of cell label for 30% of the dataset(s) based on the labels of the 50 nearest neighbors. (**b**) Distributions of cell type inter- and intra-type distances for the ambient or reduced space (bottom). K-S distance shown as measure of separation, where higher values denote greater separation (see Methods in S1 Text).

Beyond the use of dimensionality reduction to “validate” dataset merging, it is common to use 2 or 3 dimensional visuals to assess appearances of clusters. This can be to justify or directly generate cluster or cell type assignments [1–3,14,15] and to infer properties of clusters (their heterogeneity, separation, or similarity) [6,13]. Such uses rely on retention of global relationships (Fig 1), where local neighbors are less important compared to maintaining group assignment or patterns of separation between groups (Fig 1). Distance preservation may also be necessary if conclusions are to be drawn on the extent of separation or locations of clusters (Fig 1). However, across datasets of various sizes [37,51], the prediction of a cell’s label (cell type or condition) based on its neighbors is consistently worse in the 2D embedding space than in higher dimensional representations, even when labels are given as with supervised UMAP (UMAP Sup.) (Fig 4A) (see Methods in S1 Text).

Each dataset where cell type was predicted (the VMH neurons, the ex and in utero E10.5 embryos, and the developing mouse brain) additionally represents different methods for cluster assignment: using different dimension reduction and iterative clustering methods with manual selection and curation of certain cell types (for the VMH and developing brain datasets) [37,51], differential expression/enrichment analysis of marker expression to assign selected cells (in all 3), and prediction of tissue assignment from gene module expression (E10.5 dataset) [8]. Cell condition, for the 96-plex NSCs, was determined from sequencing of the multiplexing barcodes, orthogonal to analysis of the gene expression matrix. For the VMH and developing brain datasets, cell type prediction was also tested on PCA (or hierarchical Poisson factorization, HPF)-preprocessed embeddings more closely resembling embeddings used for the original assignment or visualization. These embeddings also displayed the same trends of poorer prediction once reduced to 2D (Fig M in S1 Text). Such results call into question the added benefit of using such embeddings as validations or representations of cluster assignment.

Additionally, by comparing the distribution of pairwise distances between cells of different cell “types” (“inter-type”) to the distribution of distances between cells within the same types (“intra-type”), we can measure how separated those distributions are, i.e., how separated or distinct cell types are from each other (Fig 4B) (see Methods in S1 Text). “Type” refers to either cell type (Fig 4B) or cell condition (Fig Db in S1 Text) annotations. Though it may be desirable for the low-dimensional visualizations to increase separability or clarify cell types as compared to the ambient space, such reduction can have the opposite effect (Fig 4B), reducing the gap between inter-, intra-type distributions for some datasets and increasing the gap for others, whether using the *L*_2_ or *L*_1_ norm (Figs Db, Eb, N, and O in S1 Text).

We found that cluster structures were additionally highly sensitive to the number of neighbors (perplexity for t-SNE) used in constructing nonlinear embeddings, a commonly tuned parameter which can range from 1% to 10% or less of the data [1,6], in line with other results on the effects of tuning [6,28]. For the in utero E10.5 dataset, common choices for this parameter result in different placements and overlaps of cell types, pushing progenitor populations away from their downstream cell states/types or incorrectly merging distinct, early stage populations (Fig P in S1 Text). Such inconsistencies have led to publication of incorrectly surmised differentiation trajectories from apparent relationships between cell types [52]. Even in a non-biological, machine learning, benchmark dataset [53], we found a muddling of cluster structures, with points belonging to different digits mixed within “digit-specific” clusters (possibly hidden by order of points plotted), though high accuracy classification is possible in higher dimensions [54] (Fig Q in S1 Text) (see Methods in S1 Text). This reveals an assumption of distortion cancellation in interpreting such visuals, i.e., that relevant trends will pop out despite spurious distortion/noise, and a reliance on prior knowledge of ground truth labels (or expected trends) to determine how to interpret the 2D embedding and when tuning of the esthetic parameters is sufficient.

### Density-based visuals and marker analysis

**Fig 5:**
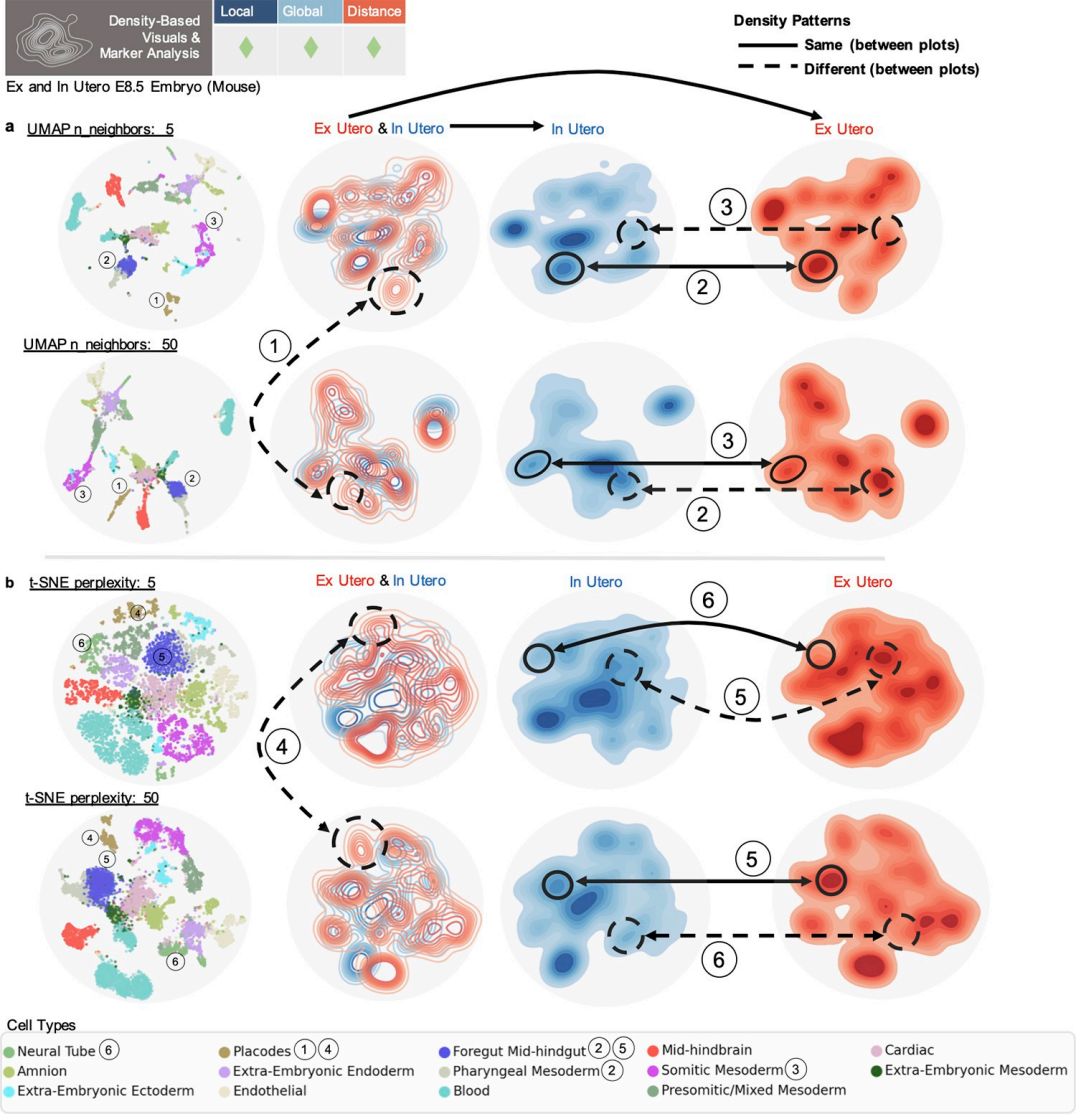
Distortion in density-based visuals and analysis. (**a**) Top row (left to right) displays UMAP embedding with n_neighbors = 5, embedding contour plot colored by condition, same contour with just in utero cells, same contour with just ex utero cells. Bottom row shows same plots for UMAP embedding with n_neighbors = 50. (**b**) Top row shows same plots for t-SNE embedding with perplexity of 5. Bottom row shows same plots for t-SNE embedding with perplexity of 50. Numbers denote comparisons between plots, dashed lines denote a difference, and solid lines denote the same appearance.

Density assessments of points in 2D embeddings are frequently used to quantitatively assess cell–cell relationships by directly relying on distances between the cells in 2 dimensions (Fig 1). Common applications compare densities of cells in different conditions or batches, within a shared embedding space, to make statements on changes in population density or expression between groups [3,16,18,20]. However, as demonstrated above, parameter tuning easily disrupts the placement of cells and clusters in such visuals, inherently affecting the generation of contours. Furthermore, using different numbers of neighbors for embedding generation can result in dramatic appearances of cell populations present in 1 condition but not the other (circled numbers 1, 4 in Fig 5A and 5B), which can disappear when more or less neighbors are used, with those populations absorbed into overlapping contours (see Methods in S1 Text). Likewise, densities of cell populations can appear of the same or different scale between conditions depending on the number of neighbors used in construction (circled numbers 2, 3, 5, 6 in Fig 5A and 5B) (Figs R and S in S1 Text), confounding the use of these visuals to make comparative statements.

### Trajectory inference and continuous relationships

**Fig 6:**
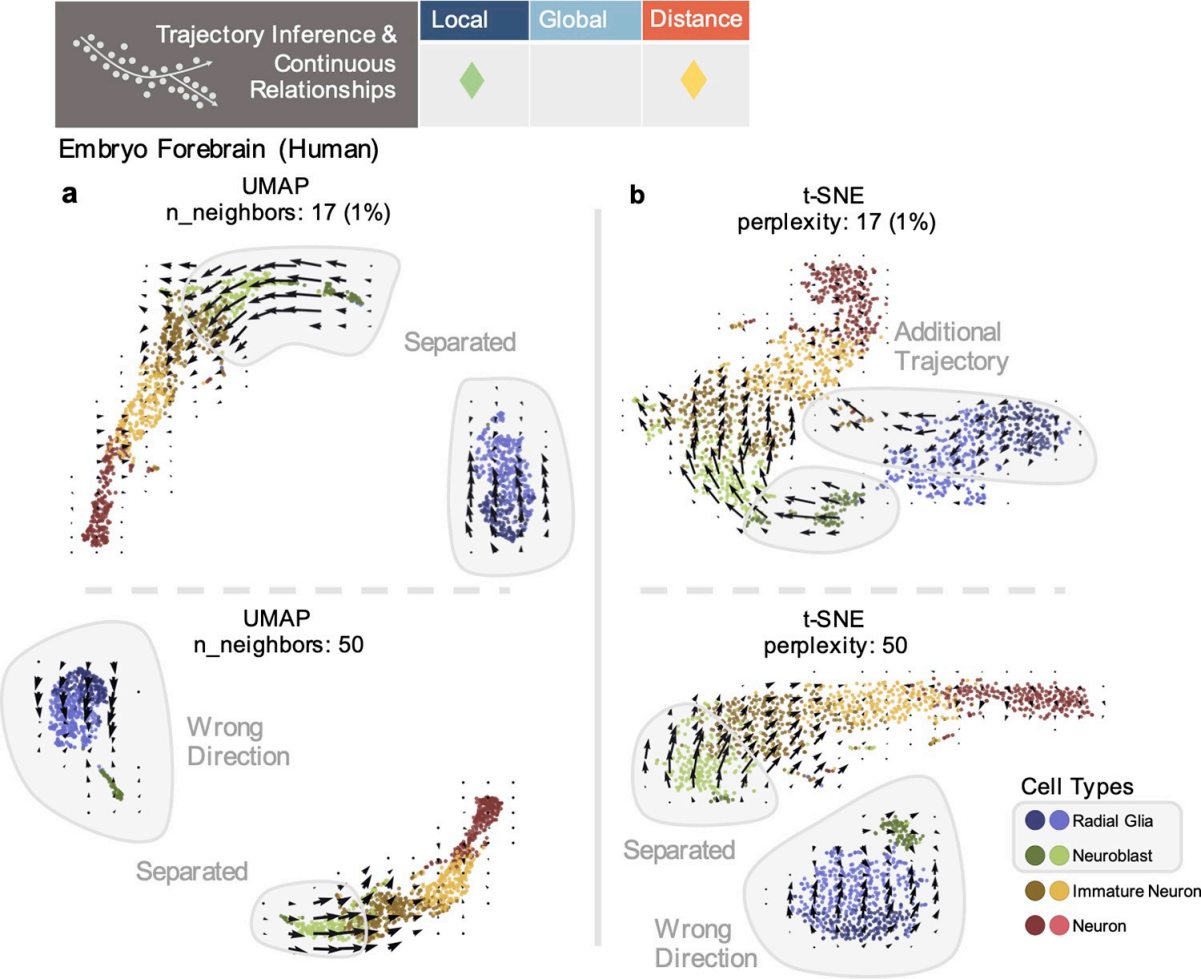
Distortion in trajectory inference and continuous relationships. (**a**) Velocyto RNA velocity embeddings for UMAPs made with 17 or 50 n_neighbors. Cell types of interest highlighted in gray. (**b**) Velocyto RNA velocity embeddings for t-SNEs made with perplexity of 17 or 50.

Trajectory inference and pseudotime tasks, such as in RNA velocity [23] or Monocle [22,24] workflows, focus on local, continuous relationships for inference and calculating pseudotime coordinates. Such tasks may also use distances between embedded points to construct the directions and magnitudes of arrows denoting inferred, developmental trajectories [23,25] (Fig 1). However, as shown with the standard velocyto workflow [23], using the neighbors of cells after reduction to 2 dimensions to construct velocity arrows can result in erroneous trajectories, due to the arbitrary placement of cells under different parameter choices. Here, we again vary the number of neighbors used to construct the embedding (see Methods in S1 Text). Distortions can include loss of continuous relationships, trajectories in incorrect directions, or the addition of new pathways for development (Fig 6) (Fig T in S1 Text). Distortions additionally occur due to upstream averaging over nearest neighbors in the inference procedure and from the choice of embedding procedure (Fig 6) [55,56]. Thus, the resulting visual compounds distortions from embedding with these prior distortive effects.

To investigate distortions of an underlying, continuous manifold by 2D reduction, we used the Swiss-roll as a non-biological benchmark dataset, for which we know the structure in 3 dimensions, and moreover is a 2D manifold (see Methods in S1 Text). We demonstrate how the 3D Swiss-roll (constructed by rolling up the 2D plane) loses its coherence when embedded in 2D with UMAP (Fig U in S1 Text). No embedding recapitulates the original plane [57] and depending on the number of neighbors used, distinct clusters or islands may appear, with a scrambling of local neighbors (made worse by increasing the tightness of the embedded roll) (Fig U in S1 Text). Thus, knowledge of the true manifold is required to understand the disruption of continuity in these embeddings.

Additionally, alongside cluster-level global relationships, locally continuous properties of such visuals are used as independent “metrics” to validate cell type assignment and robustness of clustering results [1,2,6,58]. However, in common single-cell analysis packages (e.g., Scanpy [59] and Seurat [7]), the same k-nearest neighbor (kNN) graph constructed from the higher dimensional PCA space is passed to both the clustering algorithm and the embedding algorithm. As shown in Fig V in S1 Text, the embedding is then not an independent assessment of clustering results and is likely to form clusters that resemble the kNN graph even if that graph does not represent the “original” underlying manifold. Together, the use of such embeddings to imply or infer continuous relationships then becomes an arbitrary endeavor, with a user unable to trust seemingly dramatic connections or isolated populations, and likely to choose what seems most appealing or expected.

### Embedding properties are arbitrary

To illustrate the indeterminate nature of 2D UMAP and t-SNE embeddings, we developed an autoencoder framework to fit cells from any dataset to an arbitrary 2D shape, while preserving ambient cell-to-cell distances to an extent not much different than UMAP or t-SNE (see Methods in S1 Text) [54,60,61]. We found that it is possible to embed data in the shape of a “von Neumann elephant” [62,63] or a flower. Though it is unlikely scientists would present data in such forms, as shown below, they are quantitatively similar in terms of fidelity to the data in ambient dimension, compared to UMAP or t-SNE embeddings. We call this method to produce customized embeddings “Picasso,” in homage to the eponymous artist’s skill in imitating other artistic works.

**Fig 7:**
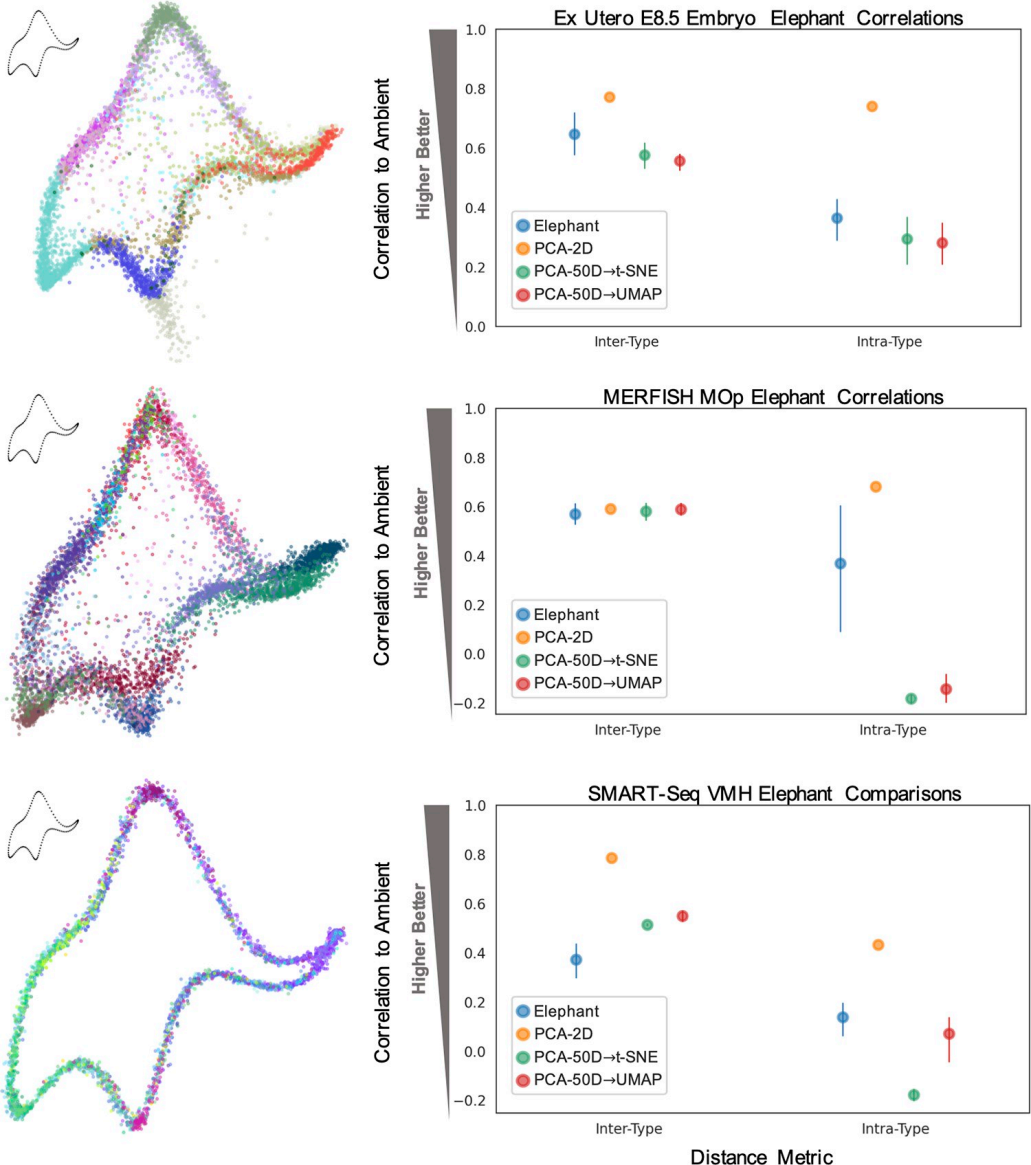
Embedding properties are arbitrary. Elephant-shaped embeddings [62,63] shown on the left, with corresponding correlations of data embeddings to ambient space shown in right-hand plots, for inter- and intra-type distance metrics. Metrics calculated over *n* = 5 embeddings. Colors denote cell types, delineated in Fig W in S1 Text.

We compared correlations of inter- and intra-type distances between Picasso embeddings with those of t-SNE, UMAP, and PCA, for the ex utero (E8.5), MERFISH MOp, and SMART-Seq VMH neuron datasets [37]. These distances represent trends often inferred from such visuals, where inter-type distances represent inter-cell-type relationships (or global relationships between clusters), and intra-type distances represent the variance or spread within the cell types (see Methods in S1 Text). Each Picasso embedding demonstrated comparable performance to t-SNE and UMAP (Fig 7), even dens-SNE/densMAP [64] projections (Fig W in S1 Text), with cells of the same types distinctly grouped together in the arbitrary shapes. Picasso embeddings also improved upon t-SNE/UMAP intra-type correlations for all datasets (Fig 7). Results were recapitulated for inter- and intra-distances calculated with the *L*_1_ norm and for trends between cells of different sexes (inter- and intra-sex distances) for the VMH neuron dataset (Figs W and X in S1 Text).

Thus, Picasso can quantitatively represent these visually inferred characteristics similarly to, or better than, the respective t-SNE/UMAP embeddings, while producing arbitrary shapes.

## Discussion

### Limitations for exploratory data analysis (EDA)

Although popular 2D embeddings can reflect the broader strokes of the data such as cell type inter-distances, or highlight correlations between features [65], our findings highlight fundamental obstacles in reduction of high-dimensional data to 2D, the generation of multiple, possibly contradictory interpretations of the same data across applications, and the limited utility of these embeddings as EDA tools.

Though at the heart of EDA, as defined by statistician John W. Tukey [66–68], is the exploration of data through visualizations prior to confirmatory analysis, such visuals are intended to encompass robust or “resistant” analyses that extract (expected or unexpected) features of the data [66]. Thus, the use of these 2D embeddings to reveal expected or unexpected properties is fraught by the fact that it is unclear which properties will be preserved or displayed, i.e., the purpose of the visual itself, where seemingly strong characteristics can be arbitrary distortions, from integration/mixing patterns (Fig 3) to the existence of or connections between clusters (Figs 4–6, Fig P in S1 Text). Methods to show error or significance of cell placement on these visuals do not tackle the inherent limitations of such low dimension embedding: the lack of definition regarding which features are displayed and what is distortion to ignore [27,69]. Prior analysis is required to determine “sufficient” tuning of esthetically oriented parameters and to define the purpose of the visual, undermining the use of such procedures as EDA tools. Together, this results in a user conducting 2 confounded exploratory analyses that of the method properties and that of the data properties.

Another of the “guiding principles” of EDA can be formulated as “analyses…before summaries” [66], where analyses are conducted to present particular features of the data, then collated as a summary. However, the use of such all-in-one visuals begins from a place of summary rather than analysis, showing “all points and all relationships” at once and attempting to approximate many properties. In general, the open-ended nature of these visuals and ability of parameter tuning to manipulate and create biological patterns demonstrate the ease with which such tools become confirmatory bias aids and that such 2D spaces should be treated more as cartoon diagrams to be displayed post-analysis. However, in these cases conceptual graphics can be used instead which do not attempt to represent “all points and all relationships” (to avoid overinterpretation) and higher-level diagrams which do not operate at the cell- or point-wise level [70,71].

### Assumptions and incoherences in the dimensionality reduction process

The generation of the 2D embedding is additionally a multistep process, demonstrated here as a preprocessing of the ambient data with a higher dimensional (linear) reduction by PCA, then a nonlinear reduction to 2D by t-SNE/UMAP. Each step incurs some distortion of the data, where preservation of certain properties by 1 reduction can be lost by the next, as well as exaggeration of distorted patterns over the steps. However, this procedure is taken as a baseline [6,28], and there is little discussion of the logic behind this coupling.

For example, though Euclidean (*L*_2_) distance is the default metric for constructing neighborhood graphs in methods such as t-SNE and UMAP, this is not a requirement, and one might surmise that the nonlinear methods instead learn other manifold-specific “metrics” from cell neighborhoods by identifying “biological geometries” (though this is not justified by the original authors [4,5]). However, methods such as UMAP and t-SNE at their core rely on measuring distances locally, in concordance with common Euclidean analysis methods. This is the case for neighborhood graph construction as used for clustering [72], pseudotime and trajectory inference [21,73], as well as nonlinear embedding (e.g., UMAP/t-SNE) [4,5]. Notably, the assumptions underlying the preprocessing of data by PCA may clash with the assumptions in extracting these other “biological geometries” by nonlinear dimensionality reduction, as PCA implicitly assumes Gaussian noise for data that lies in a Euclidean space. Embedding by PCA additionally reduces variance in the projected data, while methods such as UMAP add noise to embedded data (while removing biological signal) [74].

Utilizing these 2D visuals to infer structure of the underlying manifold then requires knowledge of that manifold itself to interpret these outputs and distortions, a task confounded by noise present in biological data and the fact that common methods poorly recapitulate simple non-Euclidean manifolds (Fig U in S1 Text) [57]. And while PCA does impose assumptions of Euclidean geometry and Gaussian noise, the assumptions of heuristic, nonlinear methods are more opaque and their results not easily falsifiable.

### Alternative methods and analysis approaches for applications

We therefore discourage reliance on and blind application of such heuristic procedures, particularly across the range of applications in Fig 1. Instead, greater focus should be given to utilizing and developing an array of investigative and self-consistent analysis tools, which provide clearer interpretation of their goals and the biological features being assessed, present targeted low-dimensional embeddings and visuals displaying these features, and can easily be combined with statistical procedures to generate and falsify hypotheses.

With respect to the general task of preserving neighbor relationships (local or global) in an embedded space, it is possible to construct embedding spaces which more explicitly control and improve nearest-neighbor structure and retention (Figs Y and Z, “MCML” in Methods in S1 Text) [75,76], as well as retention of desired metrics such as the intra-label metrics described above (Fig ZA, “bMCML” in Methods in S1 Text). However, such optimizations require making an assumption regarding the appropriate distance/similarity metric, as is generally the case with the neighborhood-based analysis methods ubiquitous across the tasks in Fig 1.

**Fig 8:**
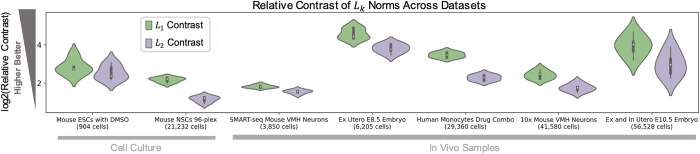
Relative contrast of the *L*_1_ and *L*_2_ metrics. Violin plots display kernel density estimates of the distribution of log2(Relative Contrast) ratio values for each dataset, computed for *n* = 5 random subsets of 1,000 HVGs selected for each dataset from its top 2,000 HVGs. Relative contrast was calculated as described in Methods in S1 Text and [30], in the ambient (gene) space. Distributions are shown across datasets of increasing sample size (cell number). Box plots are overlaid in black, with the median denoted by the white dot. Whiskers denote 1.5× the interquartile range. HVG, highly variable gene; NSC, neural stem cell; VMH, ventromedial hypothalamus.

Our analyses have focused on measuring distortions with respect to the *L*_1_ metric, given its more desirable properties in higher dimensions than Euclidean (*L*_2_) distance (see above), but other choices of distance or similarity metrics are possible and, whether in ambient or reduced space, can provide different interpretations of a dataset’s properties [33]. To assess the suitability of different metrics across datasets, we used the “relative contrast” ratio from [30] to measure the ability of an *L*_*k*_ norm to meaningfully delineate proximity between cells in high dimensions (see Methods in S1 Text). We found that *L*_1_ has higher contrast values than the *L*_2_ norm across datasets (Fig 8), suggesting preferential behavior in distinguishing cell relationships. How the various biological and technical features of each dataset drive or influence these contrast values is, however, unexplored. There are other avenues for determining the relevance of a proximity metric, by assessing data properties such as “hubness” (the presence of points with high proximity to many points in high dimensions) [77] and sparsity, discreteness, or continuity of the data structure [33], as well as the metric’s biological interpretability in light of a given task or question. Thus, if such a metric is desired to represent relationships between cells, selection of the metric(s) should be carefully considered prior to downstream transformations and dimension reductions.

Across the applications in Fig 1, there are existing methods and metrics, as well as opportunities for method development, which can provide more targeted alternatives in keeping with principles of EDA. For example, the assessment of multimodal data integration and mixing can be directly calculated between cells, as shown by the metrics in this study, as well as by other metrics on mixing proportions and separation [9] or on the retention of true “batch” differences (biological variation) [74,78]. Such analyses can additionally be conducted in the ambient space, which minimizes the distortion/transformation of gene-related properties, useful for downstream experimentation.

For applications regarding clustering, clusters can be generated from higher dimensional embeddings if not from the ambient space itself [34], and given the central importance of marker gene expression in validating cluster assignment, existing tools such as heatmaps can directly display cluster results with the features (genes) which determined these groupings. Dimensionality reduction on the gene space can additionally be used to filter for genes or sets of genes best suited to separating the clusters [79,80]. By targeting the objective of an embedding in such a manner, one can take advantage of prior knowledge/annotations and more directly determine the necessary dimensionality for a given question.

To assess heterogeneity within clusters or relationships between clusters, similarity metrics or distances can be calculated between the cells [33] and displayed with qualitative or quantitative visuals that preserve these metrics as a part of their objectives, including hierarchical relationship diagrams such as dendrograms and trees [81,82], or graph-based network diagrams [83,84]. Higher-level diagrams that do not seek to display all point-wise information can also be used to represent the results of other inter-cluster analyses [71,85], better matching the resolution of the visual to the resolution of the analysis represented.

Such cluster-level visuals and metrics, as well as metrics on integration and higher dimensional distribution comparisons as presented here, can be used in lieu of analyses based on contour plots generated from 2D coordinates. Regarding trajectories and continuous relationships, higher dimensions should be used to perform inference of differentiation trajectories [21,71], and incorporation of probabilistic and biophysically informed inference methods [70,86,87], offers falsifiable and interpretable approaches with targeted visualization alternatives. Such models additionally offer more interpretable handling of biological, as well as technical, noise [74], avoiding a smoothing over or removal of noise, which could otherwise provide valuable biological signal.

Though it may seem appealing to produce visuals of “all data and all relationships,” common embedding practice distorts data in obscure ways, attempts to pack the capabilities of many different analyses into one space, and is easily manipulated. Given these limitations, and the distortions induced by earlier processing steps [88], it is preferable to limit dimensionality reductions and ad hoc transformations, particularly when the space of interest can be treated directly, to utilize and develop targeted analyses for common questions that enable focused visuals, and collate these analyses to drive downstream, hypothesis-driven biological discovery.

## Supporting information

S1 Text Supplementary methods, figures, and note. (PDF)

## Abbreviations

EDA: exploratory data analysis
HPF: hierarchical Poisson factorization
kNN: k-nearest neighbor
mESC: mouse embryonic stem cell
NSC: neural stem cell
PCA: principal component analysis
VMH: ventromedial hypothalamus
