# Supplementary material for: Correction: The specious art of single-cell genomics
Source: PLoS Comput Biol. 2025 Nov 4;21(11):e1013655. doi: 10.1371/journal.pcbi.1013655 (PMC12585010; doi:10.1371/journal.pcbi.1013655)
Supplement: S1 File — (PDF) [file pcbi.1013655.s001.pdf]

PLOS COMPUTATIONAL BIOLOGY

PERSPECTIVE

The specious art of single-cell genomics

Tara Chari[1], **Lior Pachter**[1,2]*

1 Division of Biology and Biological Engineering, California Institute of Technology, Pasadena, California, United States of America, **2** Department of Computing and Mathematical Sciences, California Institute of Technology, Pasadena, California, United States of America

* lpachter@caltech.edu

Abstract

Dimensionality reduction is standard practice for filtering noise and identifying relevant features in large-scale data analyses. In biology, single-cell genomics studies typically begin with reduction to 2 or 3 dimensions to produce "all-in-one" visuals of the data that are amenable to the human eye, and these are subsequently used for qualitative and quantitative exploratory analysis. However, there is little theoretical support for this practice, and we show that extreme dimension reduction, from hundreds or thousands of dimensions to 2, inevitably induces significant distortion of high-dimensional datasets. We therefore examine the practical implications of low-dimensional embedding of single-cell data and find that extensive distortions and inconsistent practices make such embeddings counter-productive for exploratory, biological analyses. In lieu of this, we discuss alternative approaches for conducting targeted embedding and feature exploration to enable hypothesis-driven biological discovery.

OPEN ACCESS

Citation: Chari T, Pachter L (2023) The specious art of single-cell genomics. PLoS Comput Biol 19(8): e1011288. https://doi.org/10.1371/journal.pcbi.1011288

Editor: Jason A. Papin, University of Virginia, UNITED STATES

Published: August 17, 2023

Copyright: © 2023 Chari, Pachter. This is an open access article distributed under the terms of the Creative Commons Attribution License, which permits unrestricted use, distribution, and reproduction in any medium, provided the original author and source are credited.

Data Availability Statement: Download links for the original data used to generate the figures and results in the paper are listed in Table A in S1 Text. Processed and normalized versions of the count matrices are available on CaltechData, with links provided in Table B in S1 Text. All analysis code used to generate the figures and results in the paper is available at https://github.com/pachterlab/CP_2023 and deposited at Zenodo (DOI https://doi.org/10.5281/zenodo.8087950). Code is provided in Colab notebooks which can be run for free on the Google cloud.

Funding: L.P. received the National Institutes of Health (nih.gov) award U19MH114830, administered by the National Institute of Mental Health (nimh.nih.gov). T.C. and L.P. were partially

Introduction

The high-dimensionality of "big data" genomics datasets has led to the ubiquitous application of dimensionality reduction to filter noise, enable tractable computation, and to facilitate exploratory data analysis (EDA). Ostensibly, the goal of this reduction is to preserve and extract local and/or global structures from the data for biological inference [1–3]. Trial and error application of common techniques has resulted in a currently popular workflow combining initial dimensionality reduction to a few dozen dimensions, often using principal component analysis (PCA), with further nonlinear reduction to 2 dimensions using t-SNE [4] or UMAP [1,2,5,6]. For single-cell genomics in particular, these embeddings are used extensively in qualitative and quantitative EDA tasks that fall into 4 main categories of applications (Fig 1, "Application"):

- Modality-mixing, integration, and reference mapping:

Embeddings are used to visually assess the extent of integration, mixing, or similarities between cells from different batches [7–9] and to compare methods of integration/batch-correction [10]. For query dataset(s) mapped onto reference datasets/embeddings, visuals likewise provide an assessment of merged data similarities or differences [11,12].

- Cluster validation and relationships:

funded by this award. The funders had no role in study design, data collection and analysis, decision to publish, or preparation of the manuscript.

Competing interests: The authors have declared that no competing interests exist.

Abbreviations: EDA, exploratory data analysis; HPF, hierarchical Poisson factorization; kNN, k-nearest neighbor; mESC, mouse embryonic stem cell; NSC, neural stem cell; PCA, principal component analysis; VMH, ventromedial hypothalamus.

Visual applications range from assessing the existence of and relationships between predefined clusters, to inferring properties of the clusters (e.g., spread or heterogeneity) [1,2,13], and to generating the clusters themselves from the 2D space (e.g., to define cell types or to detect doublets) [3,14,15].

- Density-based visuals and marker analysis:

Embeddings are used to justify or measure changes in cell populations between different conditions, by comparing contour locations and sizes in the density diagrams, as well as changes in intensity or spread of gene expression [16–20].

- Trajectory inference and continuous relationships:

Embedding applications range from implying or inferring local, continuous relationships between cells and assigning pseudotime coordinates [21–24], to using the 2D coordinates for explicit calculations of magnitude and direction of developmental progression [23,25,26].

Inherent in these applications are assumptions of preservation of local and global cell properties, as well as distances, delineated in Fig 1. For each application, we demarcate which of these are the "necessary" or key geometric properties that each task inherently assumes to be represented (and preserved). Based on previous works [6,13,27,28] and the objective functions of UMAP and t-SNE [4,5], "local" is defined as nearest neighbor relationships, "global" as

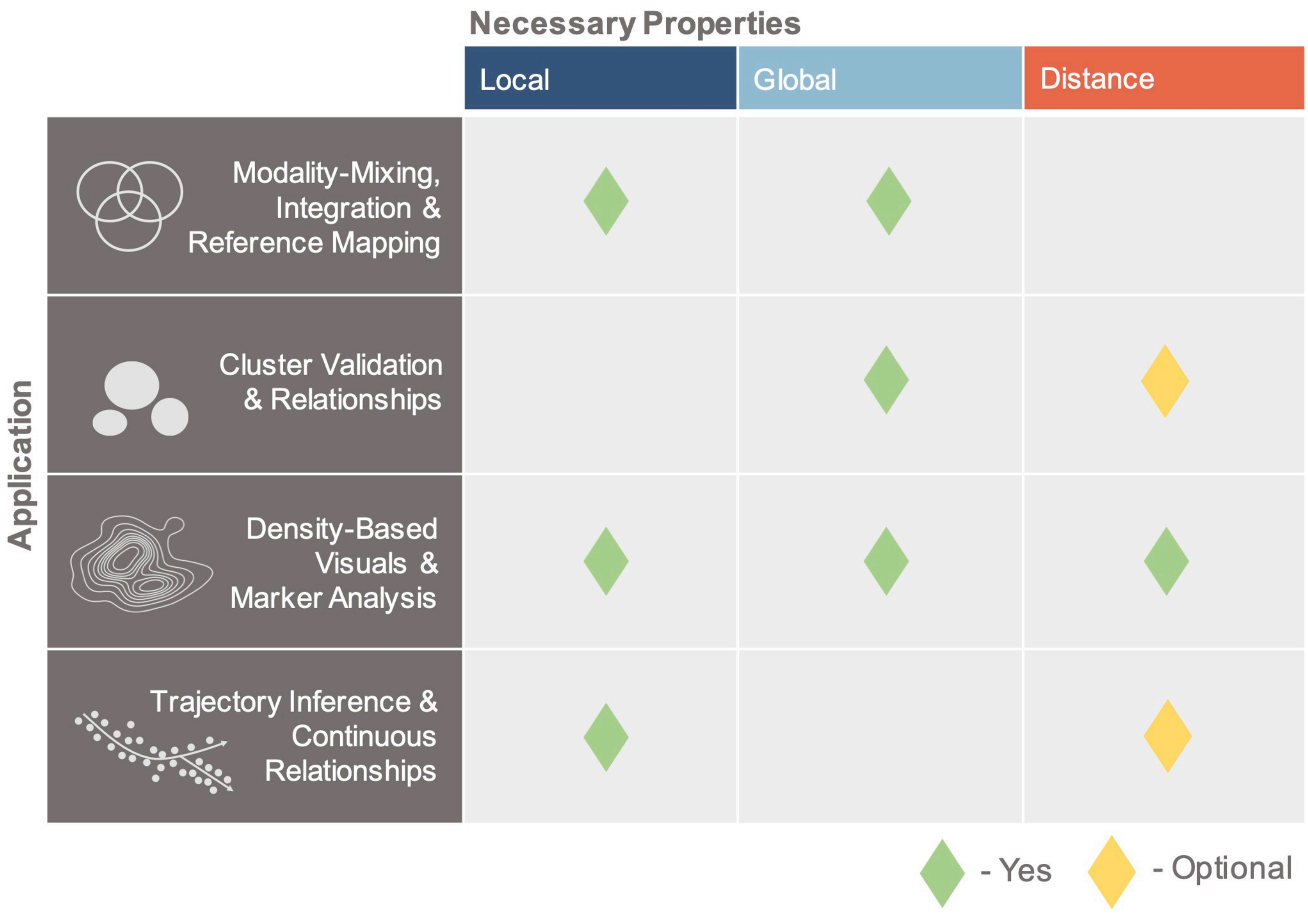

Fig 1. Necessary properties for embedding applications. Application rows denote biological tasks, and columns denote which properties are necessary, i.e., key geometric properties whose preservation or representation is assumed in the task.

https://doi.org/10.1371/journal.pcbi.1011288.g001

neighbor relationships and properties of groups of cells (e.g., cell types), and "distance" as Euclidean distance (L_2 norm) or Manhattan distance (L_1 norm) between points. Note that preservation of distance implies preservation of local and global properties. We utilize the L_2 norm as it is the default metric of UMAP/t-SNE. We also present results with the L_1 norm (see S1 Text), as L_1 is more suitable for measuring distance in high dimensions, particularly in comparison to other L_k norms [29,30], and is commonly applied to transcriptomic data [31–33], with comparable performance to the probabilistic Jensen–Shannon divergence in single-cell distance calculations [34].

Yet, despite the goals of these methods [2,3,6] to preserve local and/or global structure, there is little theory or empirical analysis to support these claims. For example, while the popular t-SNE and UMAP methods claim faithful representation of local and/or global structure in low dimensions [1,2,5], there is evidence they fail in this regard [1,35], and theorems providing guarantees on the embeddings rely on numerous assumptions unlikely to hold in practice and ignore the preprocessing by PCA prior to nonlinear reduction [36].

Here, we assess dimensionality reduction for single-cell gene expression, first investigating the preservation of the necessary properties comprising the columns of Fig 1, then assessing the impact of these embeddings across the applications comprising the rows of Fig 1.

Preservation of local and global structure in 2D embeddings

We begin with the columns of Fig 1, and assess the preservation of these properties by 2D embedding, as compared to the ambient space or higher-dimensional PCA space to which the ambient space is initially reduced prior to reduction to 2D (see Methods in S1 Text).

"Ambient" space refers to the gene count matrix after highly variable gene selection and log-normalization of the counts (see Methods in S1 Text). We denote "PCA-preprocessing" as the higher dimensional reduction of the ambient space by PCA, followed by a (nonlinear) reduction to 2D (e.g., "PCA-50D→UMAP") which mimics standard practice. Additionally, cell annotations or labels (such as cell type or condition) used in the following analyses were taken from the original studies.

Local preservation

Given the focus on preserving local nearest neighbors in the objectives of the UMAP and t-SNE methods, we first measured the recapitulation of nearest neighbors in 2D embeddings, as compared to the neighbors defined in ambient space. We used Euclidean (L_2) distance, the default for these nonlinear reduction methods, to define each cell's 30 nearest neighbors and measured Jaccard distance (dissimilarity) between the neighbors in embedding and ambient space (where 1.0 denotes no overlap). Several in vivo datasets were reduced to 2D, with PCA-preprocessing, including 10× Genomics and SMART-Seq assayed mouse ventromedial hypothalamus (VMH) neuron datasets [37], an ex utero cultured mouse embryo dataset (at the E8.5 stage) and an ex and in utero mouse embryo dataset (at the E10.5 stage) from [8], and a mouse primary motor cortex (MOp) dataset [38]. We additionally reduced cell culture-derived datasets, with and without external perturbations, including mouse embryonic stem cells (mESCs) treated in DMSO from [39] and multiplexed mouse neural stem cells (NSCs) in 96 drug combination conditions (labeled "96-plex") [40] (see Table A in S1 Text).

The 2D t-SNE/UMAP embeddings (e.g., "PCA-50D→UMAP" in Fig 2A) displayed large Jaccard distances with respect to the neighbors in ambient dimension, with an average consistently above 0.7 (70%). Generally, dissimilarity increased with the size of the dataset (Fig 2A, Figs A and Ba in S1 Text). When the number of neighbors (k), considered in the dissimiliarity calculation, was varied between 5 to 100, smaller dataset embeddings displayed slightly

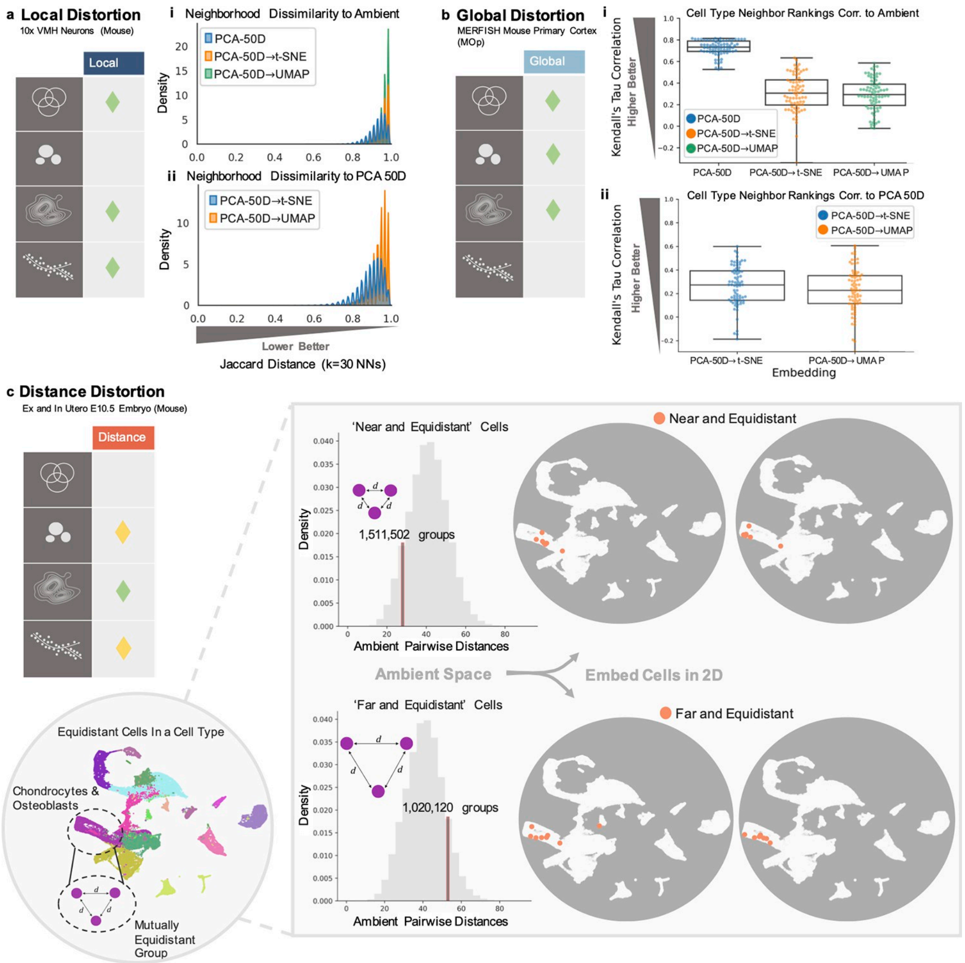

Fig 2. Distortion of necessary properties in embeddings. (**a**) (i) Distribution of Jaccard distance of cell neighbors in PCA-preprocessed 2D embeddings and the relevant PCA space, as compared to ambient space. (ii) Distribution of Jaccard distance of cell neighbors in PCA-preprocessed 2D embeddings, as compared to the higher dimensional PCA space. (**b**) (i) Boxplot of correlations of cell type neighbor rankings to ambient space for the PCA-preprocessed 2D embeddings and the relevant PCA space. (ii) Boxplot of correlations of cell type neighbor rankings to the relevant higher dimensional PCA space for the PCA-preprocessed 2D embeddings. Embeddings generated $n = 3$ times. (**c**) Selection of equidistant groups with "near" or "far" distances in ambient space. UMAP embedding of the data in gray circles, with orange circles denoting all cells within the previously determined equidistant groups.

https://doi.org/10.1371/journal.pcbi.1011288.g002

improved dissimilarity scores with larger k (Figs Bb and Bc in S1 Text). Interestingly, the embeddings of the more homogeneous mESCs dataset displayed relatively higher dissimilarity despite the small number of cells (Figs Bb and Bc in S1 Text). Poor neighborhood overlap was additionally retained, and often worsened, without PCA-preprocessing (i.e., direct reduction to 2D from ambient space). In some cases, the dissimilarity of neighbors was worse for 2D PCA ("PCA-2D") as compared to t-SNE or UMAP reduction without PCA-preprocessing, consistent with other findings on the poor preservation of local neighborhoods by both PCA and the nonlinear reduction methods [1,35] (Figs A and Bc in S1 Text). Similarly poor neighbor retention from the ambient space was observed in the higher dimensional PCA spaces as well ("PCA-50D" Fig 2Ai, Figs A and B in S1 Text) [35], particularly for larger datasets. Even between the PCA-preprocessed 2D embeddings and their corresponding PCA space, Jaccard distances were consistently above 0.8 on average, regardless of the dimension of the initial PCA reduction (Fig 2Aii, right panels Fig A in S1 Text).

Global preservation

Turning to global relationships, we measured the preservation of the rankings of neighbors of cell "types" rather than individual cells. Cell "types" denote either author-provided cell type (Fig 2Bii) or cell condition annotations. Rankings were constructed from average pairwise distances between the cells of the different types, across replicate 2D embeddings (see Methods in S1 Text). For the same datasets as above, and a multiplexed dataset of human monocytes treated with 40 drugs [41], correlation of cell type neighbor rankings to that of the ambient space were low (≤ 0.4) in PCA-preprocessed 2D embeddings, and at least 33% lower than those of the higher dimensional PCA spaces, with warped or even reversed correlations in comparison to the ambient (Fig 2Bi) or relevant PCA space (Fig 2Bii, Fig Ca in S1 Text). These distortions were not specific to the distance measure used; we observed similar results when using the L_1 norm to determine cell type neighbors (Fig Cb in S1 Text). This is consistent with observations made in other studies [6,28]. In general, correlation decreased over each step in the reduction process though there was not a clear trend related to other dataset properties (Figs Da and Ea in S1 Text). For analyses of recapitulation of cluster properties such as inferred heterogeneity or spread, see "Clustering validation and relationships" and "Embedding properties are arbitrary" below.

Distance preservation

To examine distance preservation, we extracted groups of cells with quantitatively distinct relationships in the ambient space of the Seurat-integrated [7] ex and in utero mouse embryo dataset (at the E10.5 stage) [8], specifically equidistant groups of cells, where the distances between cells were all either equally small ("near") or large ("far") (Fig 2C) (see Methods in S1 Text). This revealed upwards of 2.5 million such groups, with 3 to 8 cells in each (Figs Fa and Fe in S1 Text). However, once embedded into 2 dimensions, these quantitatively distinct groups of cells (orange dots on UMAPs, Fig 2C) displayed the same dispersion patterns, violating distance preservation, and rendering these distinct, transcriptomic relationships indistinguishable.

This is not surprising, given previous theoretical work on the limits of distance preservation in low dimensions, particularly for equidistant points [42–44]. The Johnson–Lindenstrauss lemma on the optimality of linear embedding [45–47] shows that preservation of pairwise distances with a margin of error of at most 20% for a modestly sized dataset of 10,000 cells would require at least 1,842 dimensions [48]. Distortion is inevitable: given n points embedded in 2 dimensions, the distortion of the ratio of their maximum distance, D, to minimum distance, d ("max/min ratio"), grows as $O(\sqrt{n})$ [49] (see Note in S1 Text).

In practice, measuring these "max/min ratios" in 2D embeddings, for the ex and in utero data (E10.5) as well as the 10× VMH neurons, revealed 4- to 200-fold increases in these ratios whether compared to the relevant PCA space or ambient space (with or without PCA-preprocessing). This was the case in groups of equidistant cells as well as groups of nearest neighbors (Figs F and G in S1 Text) and can result in trends such as displayed in Fig 2C, with cells shot out across the embedding. For both datasets, we empirically verified the growth of this distortion with the number of cells considered in each equidistant group, i.e., as more cells are considered in 2D, the distortion grows (Fig H in S1 Text). Higher dimensional PCA spaces largely maintained similar max/min ratios to the ambient space (Figs G and H in S1 Text). However, we note that in low dimensions PCA embedding of equidistant points is tantamount to applying a random projection, similarly resulting in projected points displaying numerous mirages of structure or outliers (Fig I in S1 Text).

Distortion of trends in applications

Given the distortions of the necessary properties in Fig 1, we then investigated their impact on each row or application, i.e., how in practice such embeddings affect the inferences and implications made in each application.

Modality-mixing, integration, and reference mapping

Malleability of "structure" under low-dimensional embedding is particularly apparent in the mixing properties of integrated, mapped, or batch-corrected datasets, where an integration procedure is accompanied by an embedding of the melded datasets (Fig 3, Fig J in S1 Text) [7,8]. This relies on preserving both local relationships (which cells are mixed) and global patterns (overall trends of mixing or non-mixing between datasets) (Fig 1). For the integrated ex and in utero dataset (E10.5), we calculated the fraction of each cell's nearest neighbors with the same label as the cell, to compare whether embeddings accurately reflect the extent of mixing of ex and in utero cells by integration (Fig 3A) (see Methods in S1 Text).

The "Log-Normalized" integrated, ambient data displayed a largely unimodal, well-mixed distribution of cells between conditions, while the distribution generated from embedding into 2 dimensions was shifted towards unmixed (left side, Fig 3A). The "Variance-Stabilized

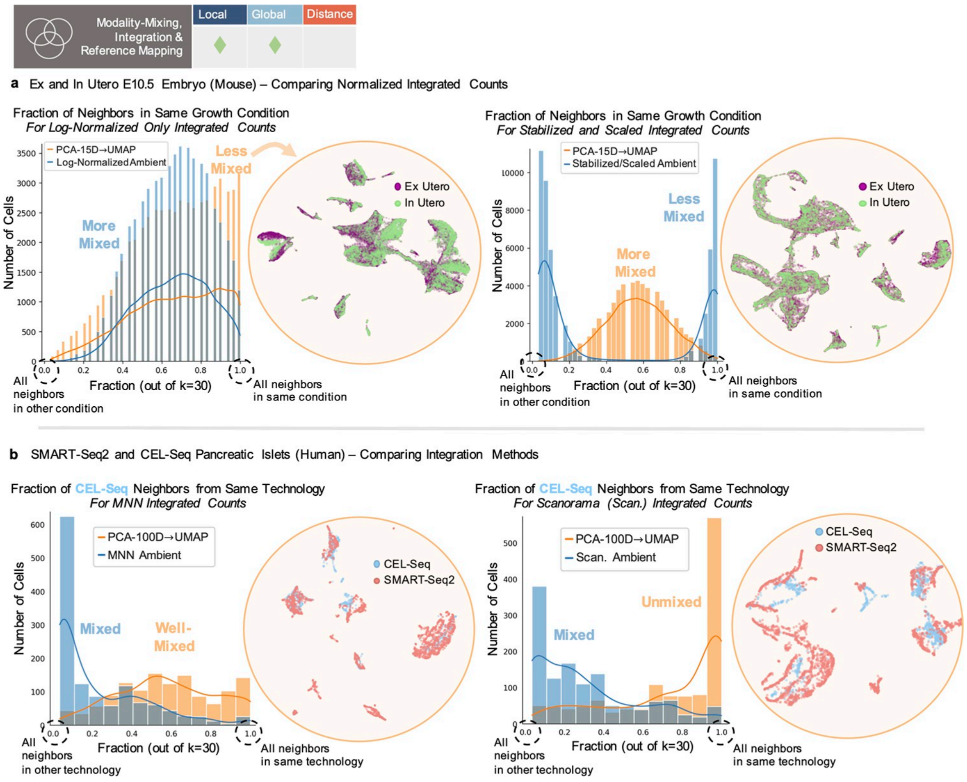

Fig 3. Distortion of mixing patterns. (**a**) Left plot shows "Log-normalized" ambient (blue) and 2D embedding (orange) distributions of mixing (fraction of cell neighbors in the same condition), where 1.0 is no mixing. Corresponding UMAP shown next to it. Right plot shows "Variance-Stabilized and Scaled" ambient (blue) and 2D embedding (orange) distributions of mixing (fraction of cell neighbors in the same condition). Corresponding UMAP shown next to it. (**b**) Left plot shows "MNN Integrated" ambient (blue) and 2D embedding (orange) distributions of mixing (fraction of cell neighbors in the same condition) for CEL-Seq cells. Corresponding UMAP shown next to it. Right plot shows "Scanorama Integrated" ambient (blue) and 2D embedding (orange) distributions of mixing (fraction of cell neighbors in the same condition) for CEL-Seq cells. Corresponding UMAP shown next to it.

https://doi.org/10.1371/journal.pcbi.1011288.g003

and Scaled" integrated, ambient data (a separate scaling procedure performed after integration) displayed the opposite trend. The ambient data presented a bimodal distribution with completely unmixed cell populations, while the final embedding displayed a unimodal distribution of well-mixed cells from both conditions (right side, Fig 3A). These additions or losses of mixing properties by 2D embedding were replicated using the L_1 metric for neighbor determination (Fig J in S1 Text).

Such mixing patterns are not only used to argue that different datasets are similar, but also to argue for the superiority of one integration method over another. To assess whether such inferences are legitimate, we merged the SMART-Seq2 and CEL-Seq pancreatic islet datasets utilized in [10] with one of 2 methods, MNN [50] or Scanorama [10]. Looking at the fraction of mixing of CEL-Seq cells in the merged ambient space reveals similar mixing by both methods (CEL-Seq cells "mapped" to SMART-Seq2 cells) (ambient distributions, Fig 3B). However the UMAP embeddings provide opposite pictures, with MNN appearing to result in a well-mixed distribution of CEL-Seq cells (left side, Fig 3B) and Scanorama an unmixed distribution of cells (right side, Fig 3B). In cases where batch correction largely fails (Fig Kb in S1 Text), the "integrated" ambient spaces (by either method) are similar to the pre-integrated ambient space. However, reduction to 2D can enhance mixing for the "integrated" spaces, but decrease mixing in the pre-integrated space. We found similar distortions when the L_1 norm was used and with t-SNE as used in [10] (Figs Jb, Jc, and Ka in S1 Text). Notably, the initial PCA reduction can drive the reversal or distortion of mixing trends, though removal of PCA-preprocessing does not alleviate this issue (Figs Jc and Ka in S1 Text). Thus, for a user, it is unclear what patterns of mixing are a result of the efficacy of the integration method, or arbitrary variation introduced by the dimensionality reduction procedure.

A consequence of these findings is that reference mapping procedures, which aim to demonstrate shared structures between batches or datasets, can also result in appearance of false structures (Fig L in S1 Text). As an example, UMAP has been proposed as a method for transforming or mapping new data given coordinates fit on another dataset [11]. Yet, transforming high dimensional, uniformly distributed points with UMAP coordinates from a single-cell dataset imposes a false structure akin to the structure of the single-cell data (Fig L in S1 Text) (see Methods in S1 Text).

Cluster validation and relationships

Beyond the use of dimensionality reduction to "validate" dataset merging, it is common to use 2 or 3 dimensional visuals to assess appearances of clusters. This can be to justify or directly generate cluster or cell type assignments [1–3,14,15] and to infer properties of clusters (their heterogeneity, separation, or similarity) [6,13]. Such uses rely on retention of global relationships (Fig 1), where local neighbors are less important compared to maintaining group assignment or patterns of separation between groups (Fig 1). Distance preservation may also be necessary if conclusions are to be drawn on the extent of separation or locations of clusters (Fig 1). However, across datasets of various sizes [37,51], the prediction of a cell's label (cell type or condition) based on its neighbors is consistently worse in the 2D embedding space than in higher dimensional representations, even when labels are given as with supervised UMAP (UMAP Sup.) (Fig 4A) (see Methods in S1 Text).

Each dataset where cell type was predicted (the VMH neurons, the ex and in utero E10.5 embryos, and the developing mouse brain) additionally represents different methods for cluster assignment: using different dimension reduction and iterative clustering methods with manual selection and curation of certain cell types (for the VMH and developing brain datasets) [37,51], differential expression/enrichment analysis of marker expression to assign

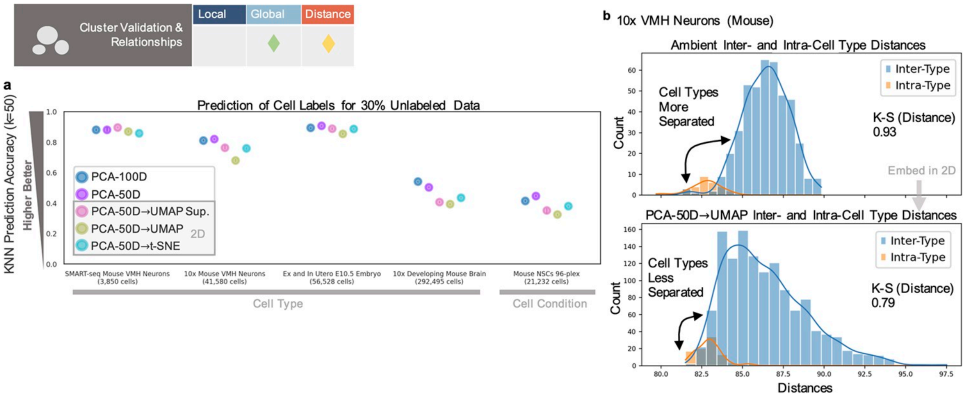

Fig 4. Distortion in cluster validation and relationships. (**a**) Prediction of cell label for 30% of the dataset(s) based on the labels of the 50 nearest neighbors. (**b**) Distributions of cell type inter- and intra-type distances for the ambient or reduced space (bottom). K-S distance shown as measure of separation, where higher values denote greater separation (see Methods in S1 Text).

https://doi.org/10.1371/journal.pcbi.1011288.g004

selected cells (in all 3), and prediction of tissue assignment from gene module expression (E10.5 dataset) [8]. Cell condition, for the 96-plex NSCs, was determined from sequencing of the multiplexing barcodes, orthogonal to analysis of the gene expression matrix. For the VMH and developing brain datasets, cell type prediction was also tested on PCA (or hierarchical Poisson factorization, HPF)-preprocessed embeddings more closely resembling embeddings used for the original assignment or visualization. These embeddings also displayed the same trends of poorer prediction once reduced to 2D (Fig M in S1 Text). Such results call into question the added benefit of using such embeddings as validations or representations of cluster assignment.

Additionally, by comparing the distribution of pairwise distances between cells of different cell "types" ("inter-type") to the distribution of distances between cells within the same types ("intra-type"), we can measure how separated those distributions are, i.e., how separated or distinct cell types are from each other (Fig 4B) (see Methods in S1 Text). "Type" refers to either cell type (Fig 4B) or cell condition (Fig Db in S1 Text) annotations. Though it may be desirable for the low-dimensional visualizations to increase separability or clarify cell types as compared to the ambient space, such reduction can have the opposite effect (Fig 4B), reducing the gap between inter-, intra-type distributions for some datasets and increasing the gap for others, whether using the L_2 or L_1 norm (Figs Db, Eb, N, and O in S1 Text).

We found that cluster structures were additionally highly sensitive to the number of neighbors (perplexity for t-SNE) used in constructing nonlinear embeddings, a commonly tuned parameter which can range from 1% to 10% or less of the data [1,6], in line with other results on the effects of tuning [6,28]. For the in utero E10.5 dataset, common choices for this parameter result in different placements and overlaps of cell types, pushing progenitor populations away from their downstream cell states/types or incorrectly merging distinct, early stage populations (Fig P in S1 Text). Such inconsistencies have led to publication of incorrectly surmised differentiation trajectories from apparent relationships between cell types [52]. Even in a non-biological, machine learning, benchmark dataset [53], we found a muddling of cluster structures, with points belonging to different digits mixed within "digit-specific" clusters (possibly hidden by order of points plotted), though high accuracy classification is possible in higher dimensions [54] (Fig Q in S1 Text) (see Methods in S1 Text). This reveals an assumption of distortion cancellation in interpreting such visuals, i.e., that relevant trends will pop out despite spurious distortion/noise, and a reliance on prior knowledge of ground truth labels (or

expected trends) to determine how to interpret the 2D embedding and when tuning of the esthetic parameters is sufficient.

Density-based visuals and marker analysis

Density assessments of points in 2D embeddings are frequently used to quantitatively assess cell–cell relationships by directly relying on distances between the cells in 2 dimensions (Fig 1). Common applications compare densities of cells in different conditions or batches, within a shared embedding space, to make statements on changes in population density or expression between groups [3,16,18,20]. However, as demonstrated above, parameter tuning easily disrupts the placement of cells and clusters in such visuals, inherently affecting the generation of contours. Furthermore, using different numbers of neighbors for embedding generation can result in dramatic appearances of cell populations present in 1 condition but not the other (circled numbers 1, 4 in Fig 5A and 5B), which can disappear when more or less neighbors are used, with those populations absorbed into overlapping contours (see Methods in S1 Text). Likewise, densities of cell populations can appear of the same or different scale between conditions depending on the number of neighbors used in construction (circled numbers 2, 3, 5, 6 in Fig 5A and 5B) (Figs R and S in S1 Text), confounding the use of these visuals to make comparative statements.

Trajectory inference and continuous relationships

Trajectory inference and pseudotime tasks, such as in RNA velocity [23] or Monocle [22,24] workflows, focus on local, continuous relationships for inference and calculating pseudotime coordinates. Such tasks may also use distances between embedded points to construct the directions and magnitudes of arrows denoting inferred, developmental trajectories [23,25] (Fig 1). However, as shown with the standard velocyto workflow [23], using the neighbors of cells after reduction to 2 dimensions to construct velocity arrows can result in erroneous trajectories, due to the arbitrary placement of cells under different parameter choices. Here, we again vary the number of neighbors used to construct the embedding (see Methods in S1 Text). Distortions can include loss of continuous relationships, trajectories in incorrect directions, or the addition of new pathways for development (Fig 6) (Fig T in S1 Text). Distortions additionally occur due to upstream averaging over nearest neighbors in the inference procedure and from the choice of embedding procedure (Fig 6) [55,56]. Thus, the resulting visual compounds distortions from embedding with these prior distortive effects.

To investigate distortions of an underlying, continuous manifold by 2D reduction, we used the Swiss-roll as a non-biological benchmark dataset, for which we know the structure in 3 dimensions, and moreover is a 2D manifold (see Methods in S1 Text). We demonstrate how the 3D Swiss-roll (constructed by rolling up the 2D plane) loses its coherence when embedded in 2D with UMAP (Fig U in S1 Text). No embedding recapitulates the original plane [57] and depending on the number of neighbors used, distinct clusters or islands may appear, with a scrambling of local neighbors (made worse by increasing the tightness of the embedded roll) (Fig U in S1 Text). Thus, knowledge of the true manifold is required to understand the disruption of continuity in these embeddings.

Additionally, alongside cluster-level global relationships, locally continuous properties of such visuals are used as independent "metrics" to validate cell type assignment and robustness of clustering results [1,2,6,58]. However, in common single-cell analysis packages (e.g., Scanpy [59] and Seurat [7]), the same k-nearest neighbor (kNN) graph constructed from the higher dimensional PCA space is passed to both the clustering algorithm and the embedding algorithm. As shown in Fig V in S1 Text, the embedding is then not an independent assessment of

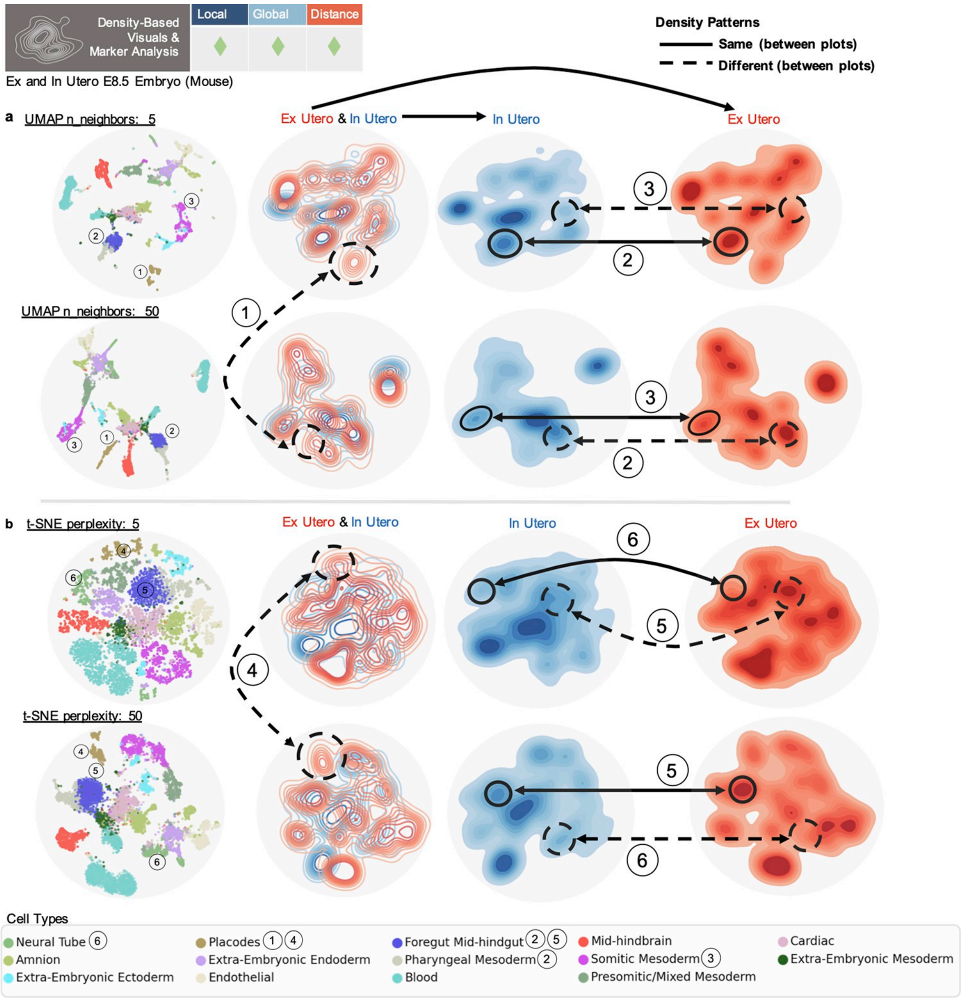

Fig 5. Distortion in density-based visuals and analysis. (**a**) Top row (left to right) displays UMAP embedding with n_neighbors = 5, embedding contour plot colored by condition, same contour with just in utero cells, same contour with just ex utero cells. Bottom row shows same plots for UMAP embedding with n_neighbors = 50. (**b**) Top row shows same plots for t-SNE embedding with perplexity of 5. Bottom row shows same plots for t-SNE embedding with perplexity of 50. Numbers denote comparisons between plots, dashed lines denote a difference, and solid lines denote the same appearance.

https://doi.org/10.1371/journal.pcbi.1011288.g005

clustering results and is likely to form clusters that resemble the kNN graph even if that graph does not represent the "original" underlying manifold. Together, the use of such embeddings to imply or infer continuous relationships then becomes an arbitrary endeavor, with a user unable to trust seemingly dramatic connections or isolated populations, and likely to choose what seems most appealing or expected.

Embedding properties are arbitrary

To illustrate the indeterminate nature of 2D UMAP and t-SNE embeddings, we developed an autoencoder framework to fit cells from any dataset to an arbitrary 2D shape, while preserving ambient cell-to-cell distances to an extent not much different than UMAP or t-SNE (see Methods in S1 Text) [54,60,61]. We found that it is possible to embed data in the shape of a "von Neumann elephant" [62,63] or a flower. Though it is unlikely scientists would present data in such forms, as shown below, they are quantitatively similar in terms of fidelity to the data in

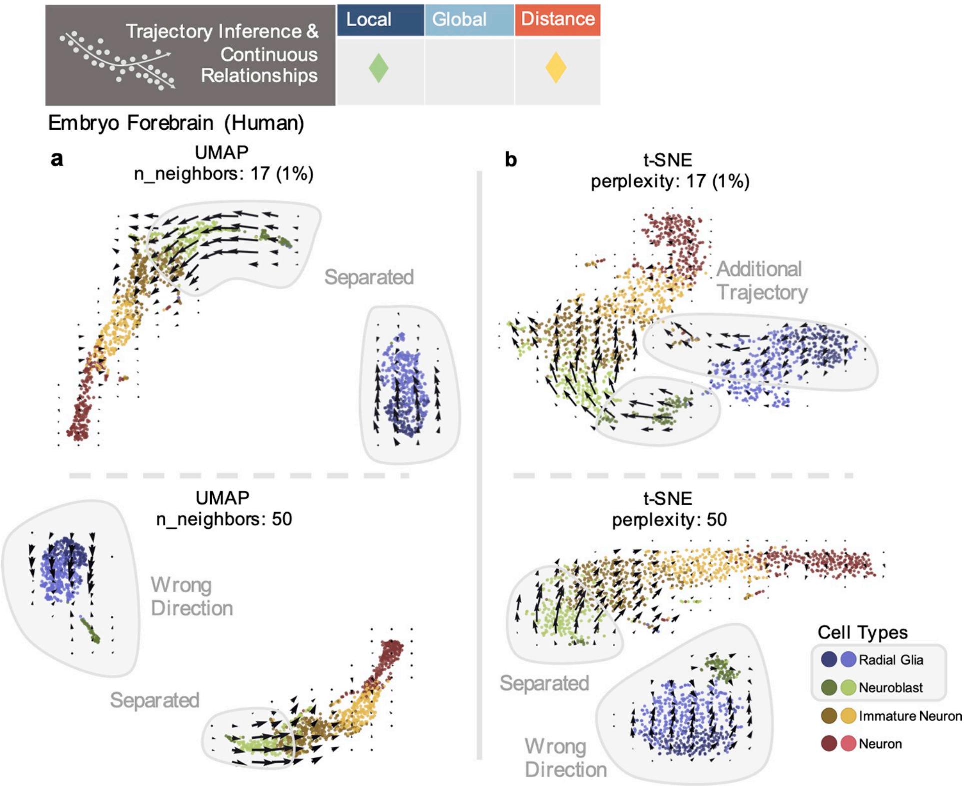

Fig 6. Distortion in trajectory inference and continuous relationships. (**a**) Velocyto RNA velocity embeddings for UMAPs made with 17 or 50 n_neighbors. Cell types of interest highlighted in gray. (**b**) Velocyto RNA velocity embeddings for t-SNEs made with perplexity of 17 or 50.

https://doi.org/10.1371/journal.pcbi.1011288.g006

ambient dimension, compared to UMAP or t-SNE embeddings. We call this method to produce customized embeddings "Picasso," in homage to the eponymous artist's skill in imitating other artistic works.

We compared correlations of inter- and intra-type distances between Picasso embeddings with those of t-SNE, UMAP, and PCA, for the ex utero (E8.5), MERFISH MOp, and SMART-Seq VMH neuron datasets [37]. These distances represent trends often inferred from such visuals, where inter-type distances represent inter-cell-type relationships (or global relationships between clusters), and intra-type distances represent the variance or spread within the cell types (see Methods in S1 Text). Each Picasso embedding demonstrated comparable performance to t-SNE and UMAP (Fig 7), even dens-SNE/densMAP [64] projections (Fig W in S1 Text), with cells of the same types distinctly grouped together in the arbitrary shapes. Picasso embeddings also improved upon t-SNE/UMAP intra-type correlations for all datasets (Fig 7). Results were recapitulated for inter- and intra-distances calculated with the L_1 norm and for trends between cells of different sexes (inter- and intra-sex distances) for the VMH neuron dataset (Figs W and X in S1 Text).

Thus, Picasso can quantitatively represent these visually inferred characteristics similarly to, or better than, the respective t-SNE/UMAP embeddings, while producing arbitrary shapes.

Discussion

Limitations for exploratory data analysis (EDA)

Although popular 2D embeddings can reflect the broader strokes of the data such as cell type inter-distances, or highlight correlations between features [65], our findings highlight

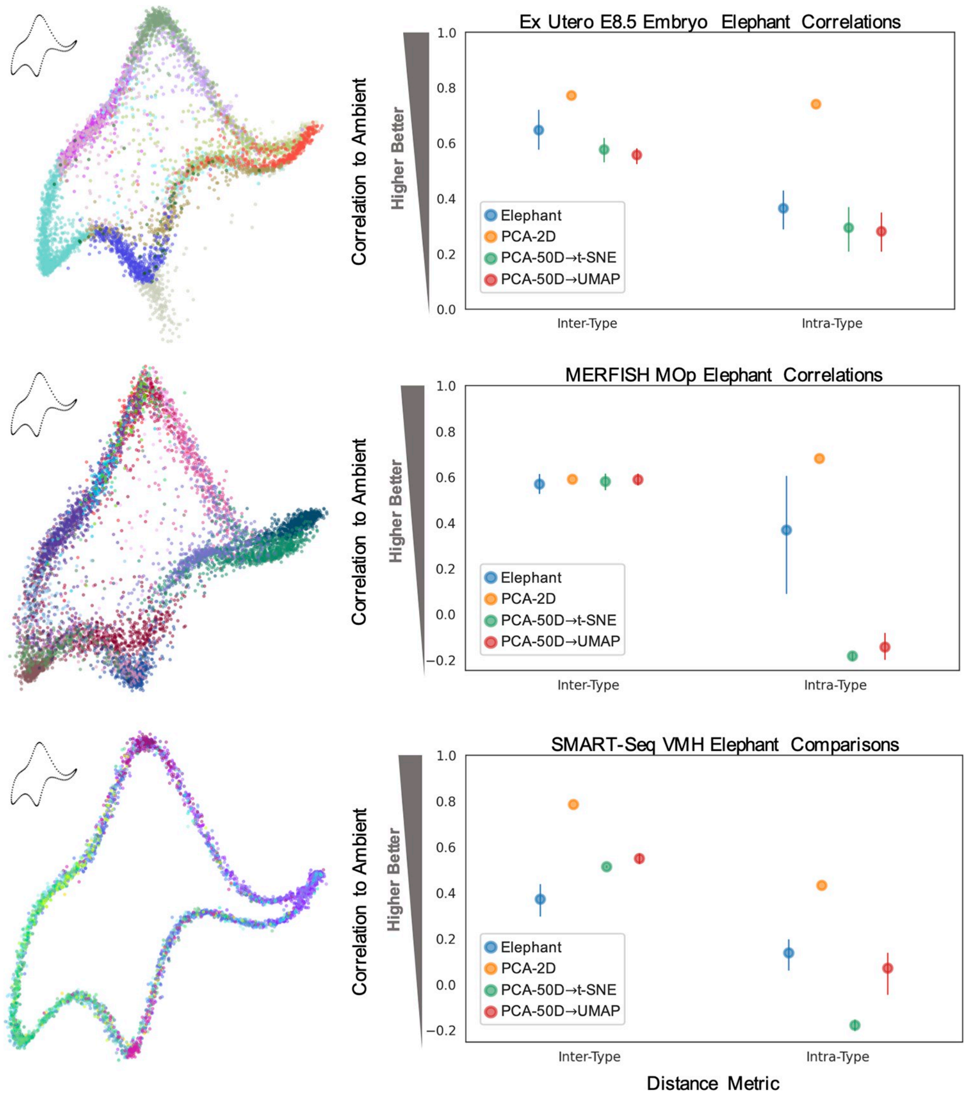

Fig 7. Embedding properties are arbitrary. Elephant-shaped embeddings [62,63] shown on the left, with corresponding correlations of data embeddings to ambient space shown in right-hand plots, for inter- and intra-type distance metrics. Metrics calculated over $n = 5$ embeddings. Colors denote cell types, delineated in Fig W in S1 Text.

https://doi.org/10.1371/journal.pcbi.1011288.g007

fundamental obstacles in reduction of high-dimensional data to 2D, the generation of multiple, possibly contradictory interpretations of the same data across applications, and the limited utility of these embeddings as EDA tools.

Though at the heart of EDA, as defined by statistician John W. Tukey [66–68], is the exploration of data through visualizations prior to confirmatory analysis, such visuals are intended to encompass robust or "resistant" analyses that extract (expected or unexpected) features of the data [66]. Thus, the use of these 2D embeddings to reveal expected or unexpected properties is fraught by the fact that it is unclear which properties will be preserved or displayed, i.e., the purpose of the visual itself, where seemingly strong characteristics can be arbitrary distortions, from integration/mixing patterns (Fig 3) to the existence of or connections between clusters (Figs 4–6, Fig P in S1 Text). Methods to show error or significance of cell placement on these visuals do not tackle the inherent limitations of such low dimension embedding: the

lack of definition regarding which features are displayed and what is distortion to ignore [27,69]. Prior analysis is required to determine "sufficient" tuning of esthetically oriented parameters and to define the purpose of the visual, undermining the use of such procedures as EDA tools. Together, this results in a user conducting 2 confounded exploratory analyses that of the method properties and that of the data properties.

Another of the "guiding principles" of EDA can be formulated as "analyses. . .before summaries" [66], where analyses are conducted to present particular features of the data, then collated as a summary. However, the use of such all-in-one visuals begins from a place of summary rather than analysis, showing "all points and all relationships" at once and attempting to approximate many properties. In general, the open-ended nature of these visuals and ability of parameter tuning to manipulate and create biological patterns demonstrate the ease with which such tools become confirmatory bias aids and that such 2D spaces should be treated more as cartoon diagrams to be displayed post-analysis. However, in these cases conceptual graphics can be used instead which do not attempt to represent "all points and all relationships" (to avoid overinterpretation) and higher-level diagrams which do not operate at the cell- or point-wise level [70,71].

Assumptions and incoherences in the dimensionality reduction process

The generation of the 2D embedding is additionally a multistep process, demonstrated here as a preprocessing of the ambient data with a higher dimensional (linear) reduction by PCA, then a nonlinear reduction to 2D by t-SNE/UMAP. Each step incurs some distortion of the data, where preservation of certain properties by 1 reduction can be lost by the next, as well as exaggeration of distorted patterns over the steps. However, this procedure is taken as a baseline [6,28], and there is little discussion of the logic behind this coupling.

For example, though Euclidean (L_2) distance is the default metric for constructing neighborhood graphs in methods such as t-SNE and UMAP, this is not a requirement, and one might surmise that the nonlinear methods instead learn other manifold-specific "metrics" from cell neighborhoods by identifying "biological geometries" (though this is not justified by the original authors [4,5]). However, methods such as UMAP and t-SNE at their core rely on measuring distances locally, in concordance with common Euclidean analysis methods. This is the case for neighborhood graph construction as used for clustering [72], pseudotime and trajectory inference [21,73], as well as nonlinear embedding (e.g., UMAP/t-SNE) [4,5]. Notably, the assumptions underlying the preprocessing of data by PCA may clash with the assumptions in extracting these other "biological geometries" by nonlinear dimensionality reduction, as PCA implicitly assumes Gaussian noise for data that lies in a Euclidean space. Embedding by PCA additionally reduces variance in the projected data, while methods such as UMAP add noise to embedded data (while removing biological signal) [74].

Utilizing these 2D visuals to infer structure of the underlying manifold then requires knowledge of that manifold itself to interpret these outputs and distortions, a task confounded by noise present in biological data and the fact that common methods poorly recapitulate simple non-Euclidean manifolds (Fig U in S1 Text) [57]. And while PCA does impose assumptions of Euclidean geometry and Gaussian noise, the assumptions of heuristic, nonlinear methods are more opaque and their results not easily falsifiable.

Alternative methods and analysis approaches for applications

We therefore discourage reliance on and blind application of such heuristic procedures, particularly across the range of applications in Fig 1. Instead, greater focus should be given to utilizing and developing an array of investigative and self-consistent analysis tools, which provide

clearer interpretation of their goals and the biological features being assessed, present targeted low-dimensional embeddings and visuals displaying these features, and can easily be combined with statistical procedures to generate and falsify hypotheses.

With respect to the general task of preserving neighbor relationships (local or global) in an embedded space, it is possible to construct embedding spaces which more explicitly control and improve nearest-neighbor structure and retention (Figs Y and Z, "MCML" in Methods in S1 Text) [75,76], as well as retention of desired metrics such as the intra-label metrics described above (Fig ZA, "bMCML" in Methods in S1 Text). However, such optimizations require making an assumption regarding the appropriate distance/similarity metric, as is generally the case with the neighborhood-based analysis methods ubiquitous across the tasks in Fig 1.

Our analyses have focused on measuring distortions with respect to the L_1 metric, given its more desirable properties in higher dimensions than Euclidean (L_2) distance (see above), but other choices of distance or similarity metrics are possible and, whether in ambient or reduced space, can provide different interpretations of a dataset's properties [33]. To assess the suitability of different metrics across datasets, we used the "relative contrast" ratio from [30] to measure the ability of an L_k norm to meaningfully delineate proximity between cells in high dimensions (see Methods in S1 Text). We found that L_1 has higher contrast values than the L_2 norm across datasets (Fig 8), suggesting preferential behavior in distinguishing cell relationships. How the various biological and technical features of each dataset drive or influence these contrast values is, however, unexplored. There are other avenues for determining the relevance of a proximity metric, by assessing data properties such as "hubness" (the presence of points with high proximity to many points in high dimensions) [77] and sparsity, discreteness, or continuity of the data structure [33], as well as the metric's biological interpretability in light of a given task or question. Thus, if such a metric is desired to represent relationships between cells, selection of the metric(s) should be carefully considered prior to downstream transformations and dimension reductions.

Across the applications in Fig 1, there are existing methods and metrics, as well as opportunities for method development, which can provide more targeted alternatives in keeping with principles of EDA. For example, the assessment of multimodal data integration and mixing can be directly calculated between cells, as shown by the metrics in this study, as well as by other metrics on mixing proportions and separation [9] or on the retention of true "batch" differences (biological variation) [74,78]. Such analyses can additionally be conducted in the ambient space, which minimizes the distortion/transformation of gene-related properties, useful for downstream experimentation.

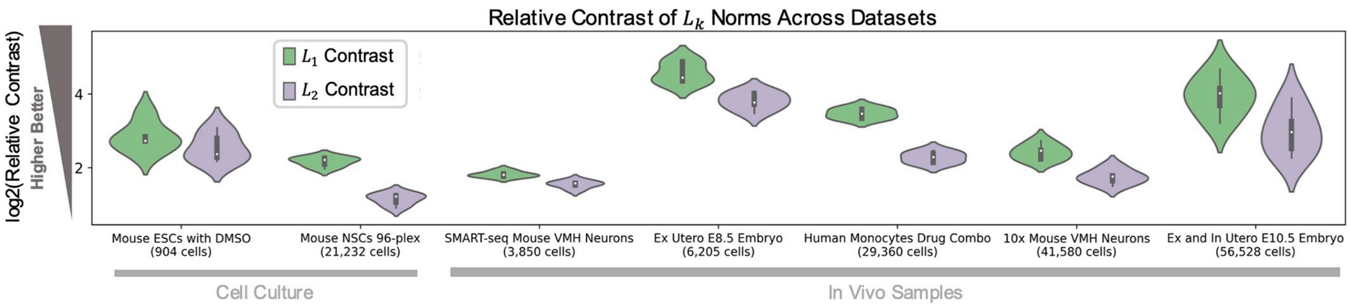

Fig 8. Relative contrast of the L_1 and L_2 metrics. Violin plots display kernel density estimates of the distribution of log2(Relative Contrast) ratio values for each dataset, computed for $n = 5$ random subsets of 1,000 HVGs selected for each dataset from its top 2,000 HVGs. Relative contrast was calculated as described in Methods in S1 Text and [30], in the ambient (gene) space. Distributions are shown across datasets of increasing sample size (cell number). Box plots are overlaid in black, with the median denoted by the white dot. Whiskers denote 1.5× the interquartile range. HVG, highly variable gene; NSC, neural stem cell; VMH, ventromedial hypothalamus.

https://doi.org/10.1371/journal.pcbi.1011288.g008

For applications regarding clustering, clusters can be generated from higher dimensional embeddings if not from the ambient space itself [34], and given the central importance of marker gene expression in validating cluster assignment, existing tools such as heatmaps can directly display cluster results with the features (genes) which determined these groupings. Dimensionality reduction on the gene space can additionally be used to filter for genes or sets of genes best suited to separating the clusters [79,80]. By targeting the objective of an embedding in such a manner, one can take advantage of prior knowledge/annotations and more directly determine the necessary dimensionality for a given question.

To assess heterogeneity within clusters or relationships between clusters, similarity metrics or distances can be calculated between the cells [33] and displayed with qualitative or quantitative visuals that preserve these metrics as a part of their objectives, including hierarchical relationship diagrams such as dendrograms and trees [81,82], or graph-based network diagrams [83,84]. Higher-level diagrams that do not seek to display all point-wise information can also be used to represent the results of other inter-cluster analyses [71,85], better matching the resolution of the visual to the resolution of the analysis represented.

Such cluster-level visuals and metrics, as well as metrics on integration and higher dimensional distribution comparisons as presented here, can be used in lieu of analyses based on contour plots generated from 2D coordinates. Regarding trajectories and continuous relationships, higher dimensions should be used to perform inference of differentiation trajectories [21,71], and incorporation of probabilistic and biophysically informed inference methods [70,86,87], offers falsifiable and interpretable approaches with targeted visualization alternatives. Such models additionally offer more interpretable handling of biological, as well as technical, noise [74], avoiding a smoothing over or removal of noise, which could otherwise provide valuable biological signal.

Though it may seem appealing to produce visuals of "all data and all relationships," common embedding practice distorts data in obscure ways, attempts to pack the capabilities of many different analyses into one space, and is easily manipulated. Given these limitations, and the distortions induced by earlier processing steps [88], it is preferable to limit dimensionality reductions and ad hoc transformations, particularly when the space of interest can be treated directly, to utilize and develop targeted analyses for common questions that enable focused visuals, and collate these analyses to drive downstream, hypothesis-driven biological discovery.

Supporting information

S1 Text. Supplementary methods, figures, and note.
(PDF)

Acknowledgments

Some of the computations presented here were conducted using machines in the Resnick High Performance Center, a facility supported by the Resnick Sustainability Institute at the California Institute of Technology. We thank Joeyta Banerjee for her work developing data processing scripts and Colab notebooks for a previous manuscript version [89]. We also thank Gennady Gorin and Benjamin Rivière for helpful discussions regarding the MCML and Picasso analyses, Sina Booeshaghi for helpful discussions regarding NCA and dimensionality reduction, Ingileif Hallgrímsdottir for valuable feedback on the manuscript, and Páll Melsted for useful insights regarding Theorem 1.

Author Contributions

Conceptualization: Tara Chari, Lior Pachter.

Data curation: Tara Chari.

Formal analysis: Tara Chari.

Funding acquisition: Lior Pachter.

Investigation: Tara Chari.

Methodology: Tara Chari.

Project administration: Tara Chari, Lior Pachter.

Resources: Lior Pachter.

Software: Tara Chari.

Supervision: Lior Pachter.

Validation: Tara Chari.

Visualization: Tara Chari.

Writing – original draft: Tara Chari, Lior Pachter.

Writing – review & editing: Tara Chari, Lior Pachter.

References

1. Kobak D, Berens P. The art of using t-SNE for single-cell transcriptomics. Nat Commun. 2019; 10 (1):5416. https://doi.org/10.1038/s41467-019-13056-x PMID: 31780648
2. Kharchenko PV. The triumphs and limitations of computational methods for scRNA-seq. Nat Methods. 2021; 18(7):723–732. https://doi.org/10.1038/s41592-021-01171-x PMID: 34155396
3. Yang Y, Sun H, Zhang Y, Zhang T, Gong J, Wei Y, et al. Dimensionality reduction by UMAP reinforces sample heterogeneity analysis in bulk transcriptomic data. Cell Rep. 2021; 36(4):109442. https://doi.org/10.1016/j.celrep.2021.109442 PMID: 34320340
4. van der Maaten L, Hinton G. Visualizing Data using t-SNE. J Mach Learn Res. 2008; 9(86):2579–2260.
5. McInnes L, Healy J, Melville J. UMAP: Uniform Manifold Approximation and Projection for Dimension Reduction. arXiv. 2018.
6. Heiser CN, Lau KS. A Quantitative Framework for Evaluating Single-Cell Data Structure Preservation by Dimensionality Reduction Techniques. Cell Rep. 2020; 31(5):107576. https://doi.org/10.1016/j.celrep.2020.107576 PMID: 32375029
7. Hao Y, Hao S, Andersen-Nissen E, Mauck WM 3rd, Zheng S, Butler A, et al. Integrated analysis of multimodal single-cell data. Cell. 2021. https://doi.org/10.1016/j.cell.2021.04.048 PMID: 34062119
8. Aguilera-Castrejon A, Oldak B, Shani T, Ghanem N, Itzkovich C, Slomovich S, et al. Ex utero mouse embryogenesis from pre-gastrulation to late organogenesis. Nature. 2021; 593(7857):119–124. https://doi.org/10.1038/s41586-021-03416-3 PMID: 33731940
9. Dou J, Liang S, Mohanty V, Miao Q, Huang Y, Liang Q, et al. Bi-order multimodal integration of single-cell data. Genome Biol. 2022; 23(1):112. https://doi.org/10.1186/s13059-022-02679-x PMID: 35534898
10. Hie B, Bryson B, Berger B. Efficient integration of heterogeneous single-cell transcriptomes using Scanorama. Nat Biotechnol. 2019; 37(6):685–691. https://doi.org/10.1038/s41587-019-0113-3 PMID: 31061482
11. Becht E, McInnes L, Healy J, Dutertre CA, Kwok IWH, Ng LG, et al. Dimensionality reduction for visualizing single-cell data using UMAP. Nat Biotechnol. 2018. https://doi.org/10.1038/nbt.4314 PMID: 30531897
12. Kang JB, Nathan A, Weinand K, Zhang F, Millard N, Rumker L, et al. Efficient and precise single-cell reference atlas mapping with Symphony. Nat Commun. 2021; 12(1):5890. https://doi.org/10.1038/s41467-021-25957-x PMID: 34620862
13. Agrawal A, Ali A, Boyd S. Minimum-Distortion Embedding. arXiv. 2021.

14. DePasquale EAK, Schnell DJ, Van Camp PJ, Valiente-Alandí Í, Blaxall BC, Grimes HL, et al. DoubletDecon: Deconvoluting Doublets from Single-Cell RNA-Sequencing Data. Cell Rep. 2019; 29(6):1718–1727.e8. https://doi.org/10.1016/j.celrep.2019.09.082 PMID: 31693907
15. Peyvandipour A, Shafi A, Saberian N, Draghici S. Identification of cell types from single cell data using stable clustering. Sci Rep. 2020; 10(1):12349. https://doi.org/10.1038/s41598-020-66848-3 PMID: 32703984
16. Andreatta M, Corria-Osorio J, Müller S, Cubas R, Coukos G, Carmona SJ. Interpretation of T cell states from single-cell transcriptomics data using reference atlases. Nat Commun. 2021; 12(1):2965. https://doi.org/10.1038/s41467-021-23324-4 PMID: 34017005
17. Szabo PA, Levitin HM, Miron M, Snyder ME, Senda T, Yuan J, et al. Single-cell transcriptomics of human T cells reveals tissue and activation signatures in health and disease. Nat Commun. 2019; 10(1):4706. https://doi.org/10.1038/s41467-019-12464-3 PMID: 31624246
18. Song NJ, Allen C, Vilgelm AE, Riesenberg BP, Weller KP, Reynolds K, et al. Treatment with soluble CD24 attenuates COVID-19-associated systemic immunopathology. J Hematol Oncol. 2022; 15(1):5. https://doi.org/10.1186/s13045-021-01222-y PMID: 35012610
19. Yu H, Welch JD. PerturbNet predicts single-cell responses to unseen chemical and genetic perturbations. 2022.
20. Kamimoto K, Stringa B, Hoffmann CM, Jindal K, Solnica-Krezel L, Morris SA. Dissecting cell identity via network inference and in silico gene perturbation. Nature. 2023; 614(7949):742–751. https://doi.org/10.1038/s41586-022-05688-9 PMID: 36755098
21. Saelens W, Cannoodt R, Todorov H, Saeys Y. A comparison of single-cell trajectory inference methods. Nat Biotechnol. 2019; 37(5):547–554. https://doi.org/10.1038/s41587-019-0071-9 PMID: 30936559
22. Cao J, Spielmann M, Qiu X, Huang X, Ibrahim DM, Hill AJ, et al. The single-cell transcriptional landscape of mammalian organogenesis. Nature. 2019; 566(7745):496–502. https://doi.org/10.1038/s41586-019-0969-x PMID: 30787437
23. La Manno G, Soldatov R, Zeisel A, Braun E, Hochgerner H, Petukhov V. et al. RNA velocity of single cells. Nature. 2018; 560(7719):494–498. https://doi.org/10.1038/s41586-018-0414-6 PMID: 30089906
24. Trapnell C, Cacchiarelli D, Grimsby J, Pokharel P, Li S, Morse M, et al. The dynamics and regulators of cell fate decisions are revealed by pseudotemporal ordering of single cells. Nat Biotechnol. 2014; 32(4):381–386. https://doi.org/10.1038/nbt.2859 PMID: 24658644
25. Ma S, Zhang B, LaFave LM, Earl AS, Chiang Z, Hu Y, et al. Chromatin Potential Identified by Shared Single-Cell Profiling of RNA and Chromatin. Cell. 2020; 183(4):1103–1116.e20. https://doi.org/10.1016/j.cell.2020.09.056 PMID: 33098772
26. Hu Y, Wang X, Hu B, Mao Y, Chen Y, Yan L, et al. Dissecting the transcriptome landscape of the human fetal neural retina and retinal pigment epithelium by single-cell RNA-seq analysis. PLoS Biol. 2019; 17(7):e3000365. https://doi.org/10.1371/journal.pbio.3000365 PMID: 31269016
27. Ovchinnikova S, Anders S. Exploring dimension-reduced embeddings with Sleepwalk. Genome Res. 2020; 30(5):749–756. https://doi.org/10.1101/gr.251447.119 PMID: 32430339
28. Kobak D, Linderman GC. Initialization is critical for preserving global data structure in both t-SNE and UMAP. Nat Biotechnol. 2021; 39(2):156–157. https://doi.org/10.1038/s41587-020-00809-z PMID: 33526945
29. Batu T, Fortnow L, Rubinfeld R, Smith WD, White P. Testing that distributions are close. In: Proceedings 41st Annual Symposium on Foundations of Computer Science. Available from: ieeexplore.ieee.org; 2000. p. 259–269.
30. Aggarwal CC, Hinneburg A, Keim DA. On the Surprising Behavior of Distance Metrics in High Dimensional Space. In: Van den Bussche J, Vianu V, editors. Database Theory—ICDT 2001. Berlin, Heidelberg: Springer Berlin Heidelberg; 2001. p. 420–434.
31. Skinnider MA, Squair JW, Foster LJ. Evaluating measures of association for single-cell transcriptomics. Nat Methods. 2019; 16(5):381–386. https://doi.org/10.1038/s41592-019-0372-4 PMID: 30962620
32. Ursu O, Neal JT, Shea E, Thakore PI, Jerby-Arnon L, Nguyen L, et al. Massively parallel phenotyping of coding variants in cancer with Perturb-seq. Nat Biotechnol. 2022; 40(6):896–905. https://doi.org/10.1038/s41587-021-01160-7 PMID: 35058622
33. Watson ER, Mora A, Taherian Fard A, Mar JC. How does the structure of data impact cell–cell similarity? Evaluating how structural properties influence the performance of proximity metrics in single cell RNA-seq data. Brief Bioinform. 2022; 23(6):bbac387. https://doi.org/10.1093/bib/bbac387 PMID: 36151725
34. Ntranos V, Kamath GM, Zhang JM, Pachter L, Tse DN. Fast and accurate single-cell RNA-seq analysis by clustering of transcript-compatibility counts. Genome Biol. 2016; 17(1):112. https://doi.org/10.1186/s13059-016-0970-8 PMID: 27230763

35. Cooley SM, Hamilton T, Aragones SD, Ray JCJ, Deeds EJ. A novel metric reveals previously unrecognized distortion in dimensionality reduction of scRNA-seq data. bioRxiv. 2022. https://doi.org/10.1101/689851

36. Linderman GC, Steinerberger S. Clustering with t-SNE, Provably. SIAM J Math Data Sci. 2019; 1 (2):313–332. https://doi.org/10.1137/18m1216134 PMID: 33073204

37. Kim DW, Yao Z, Graybuck LT, Kim TK, Nguyen TN, Smith KA, et al. Multimodal Analysis of Cell Types in a Hypothalamic Node Controlling Social Behavior Analysis of Cell Types in a Hypothalamic Node Controlling Social Behavior. Cell. 2019; 179(3):713–728.e17.

38. Zhang M, Eichhorn SW, Zingg B, Yao Z, Zeng H, Dong H, et al. Molecular, spatial and projection diversity of neurons in primary motor cortex revealed by in situ single-cell transcriptomics. bioRxiv. 2020. https://doi.org/10.1101/2020.06.04.105700

39. Desai RV, Chen X, Martin B, Chaturvedi S, Hwang DW, Li W, et al. A DNA repair pathway can regulate transcriptional noise to promote cell fate transitions. Science. 2021; 373(6557). https://doi.org/10.1126/science.abc6506 PMID: 34301855

40. Gehring J, Hwee Park J, Chen S, Thomson M, Pachter L. Highly multiplexed single-cell RNA-seq by DNA oligonucleotide tagging of cellular proteins. Nat Biotechnol. 2020; 38(1):35–38. https://doi.org/10.1038/s41587-019-0372-z PMID: 31873215

41. Chen S, Rivaud P, Park JH, Tsou T, Charles E, Haliburton JR, et al. Dissecting heterogeneous cell populations across drug and disease conditions with PopAlign. Proc Natl Acad Sci U S A. 2020; 117 (46):28784–28794. https://doi.org/10.1073/pnas.2005990117 PMID: 33127759

42. Balko M, Pór A, Scheucher M, Swanepoel K, Valtr P. Almost-Equidistant Sets. Graphs Combin. 2020; 36(3):729–754.

43. Badoiu M, Dhamdhere K, Gupta A, Rabinovich Y, Räcke H, Ravi R, et al. Approximation algorithms for low-distortion embeddings into low-dimensional spaces. In: SODA. vol. 5. Citeseer; 2005. p. 119–128.

44. Matoušek J. On the distortion required for embedding finite metric spaces into normed spaces. Israel J Math. 1996; 93(1):333–344.

45. Johnson WB, Lindenstrauss J. Extensions of Lipschitz mappings into a Hilbert space 26. Contemp Math. 1984:26.

46. Larsen KG, Nelson J. The Johnson-Lindenstrauss lemma is optimal for linear dimensionality reduction. arXiv. 2014.

47. Larsen KG, Nelson J. Optimality of the Johnson-Lindenstrauss Lemma. In: 2017 IEEE 58th Annual Symposium on Foundations of Computer Science (FOCS). Available from: ieeexplore.ieee.org; 2017. p. 633–638.

48. Dasgupta S, Gupta A. An elementary proof of a theorem of Johnson and Lindenstrauss. Random Struct Algorithms. 2003; 22(1):60–65.

49. Littlewood JE. Littlewood's Miscellany. Cambridge University Press; 1986.

50. Haghverdi L, Lun ATL, Morgan MD, Marioni JC. Batch effects in single-cell RNA-sequencing data are corrected by matching mutual nearest neighbors. Nat Biotechnol. 2018; 36(5):421–427. https://doi.org/10.1038/nbt.4091 PMID: 29608177

51. La Manno G, Siletti K, Furlan A, Gyllborg D, Vinsland E. Molecular architecture of the developing mouse brain. BioRxiv. 2020.

52. Alquicira-Hernandez J, Powell JE, Phan TG. No evidence that plasmablasts transdifferentiate into developing neutrophils in severe COVID-19 disease. Clin Transl Immunology. 2021; 10(7):e1308. https://doi.org/10.1002/cti2.1308 PMID: 34221402

53. Deng L. The MNIST Database of Handwritten Digit Images for Machine Learning Research [Best of the Web]. IEEE Signal Process Mag. 2012; 29(6):141–142.

54. Byerly A, Kalganova T, Dear I. No routing needed between capsules. Neurocomputing. 2021; 463:545–553.

55. Gorin G, Fang M, Chari T, Pachter L. RNA velocity unraveled. PLoS Comput Biol. 2022; 18(9): e1010492. https://doi.org/10.1371/journal.pcbi.1010492 PMID: 36094956

56. Zheng SC, Stein-O'Brien G, Boukas L, Goff LA, Hansen KD. Pumping the brakes on RNA velocity–understanding and interpreting RNA velocity estimates. 2022.

57. Li SZ, Zang Z, Wu L. Deep Manifold Computing and Visualization. arXiv e-prints. 2020; p. arXiv–2010.

58. Ding J, Regev A. Deep generative model embedding of single-cell RNA-Seq profiles on hyperspheres and hyperbolic spaces. Nat Commun. 2021; 12(1):2554. https://doi.org/10.1038/s41467-021-22851-4 PMID: 33953202

59. Wolf FA, Angerer P, Theis FJ. SCANPY: large-scale single-cell gene expression data analysis. Genome Biol. 2018; 19(1):15. https://doi.org/10.1186/s13059-017-1382-0 PMID: 29409532

60. Svensson V, Gayoso A, Yosef N, Pachter L. Interpretable factor models of single-cell RNA-seq via variational autoencoders. Bioinformatics. 2020; 36(11):3418–3421. https://doi.org/10.1093/bioinformatics/btaa169 PMID: 32176273

61. Kingma DP, Ba J. Adam: A Method for Stochastic Optimization. arXiv. 2014.

62. Mayer J, Khairy K, Howard J. Drawing an elephant with four complex parameters. Am J Physiol. 2010; 78(6):648–649.

63. Dyson F. A meeting with Enrico Fermi. Nature. 2004; 427(6972):297. https://doi.org/10.1038/427297a PMID: 14737148

64. Narayan A, Berger B, Cho H. Assessing single-cell transcriptomic variability through density-preserving data visualization. Nat Biotechnol. 2021; 39(6):765–774. https://doi.org/10.1038/s41587-020-00801-7 PMID: 33462509

65. Dorrity MW, Saunders LM, Queitsch C, Fields S, Trapnell C. Dimensionality reduction by UMAP to visualize physical and genetic interactions. Nat Commun. 2020; 11(1):1–6.

66. Tukey JW. Exploratory data analysis as part of a larger whole. In: Proceedings of the 18th conference on design of experiments in army research and development i. Washington, DC. vol. 1010. apps.dtic.mil; 1972.

67. Tukey JW. We Need Both Exploratory and Confirmatory. Am Stat. 1980; 34(1):23–25.

68. Hoaglin DC, John W. Tukey and Data Analysis. Stat Sci. 2003; 18(3):311–318.

69. Dong X, Bacher R. Data-driven assessment of dimension reduction quality for single-cell omics data. Patterns Prejudice. 2022; 3(3):100465. https://doi.org/10.1016/j.patter.2022.100465 PMID: 35510193

70. Lin C, Bar-Joseph Z. Continuous-state HMMs for modeling time-series single-cell RNA-Seq data. Bioinformatics. 2019; 35(22):4707–4715. https://doi.org/10.1093/bioinformatics/btz296 PMID: 31038684

71. Wolf FA, Hamey FK, Plass M, Solana J, Dahlin JS, Göttgens B, et al. PAGA: graph abstraction reconciles clustering with trajectory inference through a topology preserving map of single cells. Genome Biol. 2019; 20(1):59. https://doi.org/10.1186/s13059-019-1663-x PMID: 30890159

72. De Meo P, Ferrara E, Fiumara G, Provetti A. Generalized Louvain method for community detection in large networks. In: 2011 11th International Conference on Intelligent Systems Design and Applications. Available from: ieeexplore.ieee.org; 2011. p. 88–93.

73. Haghverdi L, Büttner M, Wolf FA, Buettner F, Theis FJ. Diffusion pseudotime robustly reconstructs lineage branching. Nat Methods. 2016; 13(10):845–848. https://doi.org/10.1038/nmeth.3971 PMID: 27571553

74. Gorin G, Pachter L. Monod: mechanistic analysis of single-cell RNA sequencing count data. bioRxiv. 2022. https://doi.org/10.1101/2022.06.11.495771

75. Goldberger J, Roweis S, Hinton G, Salakhutdinov R. Neighbourhood components analysis. In: Proceedings of the 17th International Conference on Neural Information Processing Systems. NIPS'04. Cambridge, MA, USA: MIT Press; 2004. p. 513–520.

76. Xu C, Lopez R, Mehlman E, Regier J, Jordan MI, Yosef N. Probabilistic harmonization and annotation of single-cell transcriptomics data with deep generative models. Mol Syst Biol. 2021; 17(1):e9620. https://doi.org/10.15252/msb.20209620 PMID: 33491336

77. Flexer A, Schnitzer D. Choosing ℓ^p norms in high-dimensional spaces based on hub analysis. Neurocomputing. 2015; 169:281–287. https://doi.org/10.1016/j.neucom.2014.11.084 PMID: 26640321

78. Tyler SR, Bunyavanich S, Schadt EE. PMD Uncovers Widespread Cell-State Erasure by scRNAseq Batch Correction Methods. bioRxiv. 2021. https://doi.org/10.1101/2021.11.15.468733

79. Dumitrascu B, Villar S, Mixon DG, Engelhardt BE. Optimal marker gene selection for cell type discrimination in single cell analyses. Nat Commun. 2021; 12(1):1186. https://doi.org/10.1038/s41467-021-21453-4 PMID: 33608535

80. Melton S, Ramanathan S. Discovering a sparse set of pairwise discriminating features in high-dimensional data. Bioinformatics. 2021; 37(2):202–212. https://doi.org/10.1093/bioinformatics/btaa690 PMID: 32730566

81. Ribeiro-Gonçalves B, Francisco AP, Vaz C, Ramirez M, Carriço JA. PHYLOViZ Online: web-based tool for visualization, phylogenetic inference, analysis and sharing of minimum spanning trees. Nucleic Acids Res. 2016; 44(W1):W246–W251. https://doi.org/10.1093/nar/gkw359 PMID: 27131357

82. Huson DH, Kloepper T, Bryant D. SplitsTree 4.0-Computation of phylogenetic trees and networks. Bioinformatics. 2008; 14:68–73.

83. Hagberg A, Swart P, Schult D. Exploring network structure, dynamics, and function using networkx. Los Alamos National Lab. (LANL), Los Alamos, NM (United States); 2008. LA-UR-08-5495.

84. Epskamp S, Cramer AOJ, Waldorp LJ, Schmittmann VD, Borsboom D. qgraph: Network visualizations of relationships in psychometric data. J Stat Softw. 2012; 48:1–18.

85. Zhang T, Xu Y, Imai K, Fei T, Wang G, Dong B, et al. A single-cell analysis of the molecular lineage of chordate embryogenesis. Sci Adv. 2020; 6(45). https://doi.org/10.1126/sciadv.abc4773 PMID: 33148647

86. Du JH, Gao M, Wang J. Model-based Trajectory Inference for Single-Cell RNA Sequencing Using Deep Learning with a Mixture Prior. bioRxiv. 2020. https://doi.org/10.1101/2020.12.26.424452

87. Gorin G, Vastola JJ, Fang M, Pachter L. Interpretable and tractable models of transcriptional noise for the rational design of single-molecule quantification experiments. Nat Commun. 2022; 13(1):7620. https://doi.org/10.1038/s41467-022-34857-7 PMID: 36494337

88. Ahlmann-Eltze C, Huber W. Comparison of transformations for single-cell RNA-seq data. Nat Methods. 2023; 20(5):665–672. https://doi.org/10.1038/s41592-023-01814-1 PMID: 37037999

89. Chari T, Banerjee J, Pachter L. The Specious Art of Single-Cell Genomics. bioRxiv. 2021. https://doi.org/10.1101/2021.08.25.457696
